# Theory of local k-mer selection with applications to long-read alignment

**DOI:** 10.1093/bioinformatics/btab790

**Published:** 2021-11-19

**Authors:** Jim Shaw, Yun William Yu

**Affiliations:** Department of Mathematics, University of Toronto, Toronto, ON M5S 2E4, Canada; Department of Mathematics, University of Toronto, Toronto, ON M5S 2E4, Canada; Department of Computer and Mathematical Sciences, University of Toronto at Scarborough, Scarborough, ON M1C 1A4, Canada

## Abstract

**Motivation:**

Selecting a subset of k-mers in a string in a local manner is a common task in bioinformatics tools for speeding up computation. Arguably the most well-known and common method is the minimizer technique, which selects the ‘lowest-ordered’ k-mer in a sliding window. Recently, it has been shown that minimizers may be a sub-optimal method for selecting subsets of k-mers when mutations are present. There is, however, a lack of understanding behind the theory of why certain methods perform well.

**Results:**

We first theoretically investigate the conservation metric for k-mer selection methods. We derive an exact expression for calculating the conservation of a k-mer selection method. This turns out to be tractable enough for us to prove closed-form expressions for a variety of methods, including (open and closed) syncmers, (*a*, *b*, *n*)-words, and an upper bound for minimizers. As a demonstration of our results, we modified the minimap2 read aligner to use a more conserved k-mer selection method and demonstrate that there is up to an 8.2% relative increase in number of mapped reads. However, we found that the k-mers selected by more conserved methods are also more repetitive, leading to a runtime increase during alignment. We give new insight into how one might use new k-mer selection methods as a reparameterization to optimize for speed and alignment quality.

**Availability and implementation:**

Simulations and **supplementary methods** are available at https://github.com/bluenote-1577/local-kmer-selection-results. os-minimap2 is a modified version of minimap2 and available at https://github.com/bluenote-1577/os-minimap2.

**Supplementary information:**

Supplementary data are available at *Bioinformatics* online.

## 1 Introduction

In recent decades, there has been an exponential increase in the amount and throughput of available sequencing data (Goodwin *et al.*, 2016), necessitating more efficient modern methods for processing sequencing data (Berger *et al.*, 2016). Many methods employ *k-mer*- (length-*k* substrings of a sequence) based analysis, because k-mer methods tend to be fast and memory efficient. k-mer methods appear in metagenomics (Wood and Salzberg, 2014), genome assembly (Nagarajan and Pop, 2013), read alignment (Li, 2018), variant detection (Peterlongo *et al.*, 2017; Shajii *et al.*, 2016) and many more.

Because k-mers overlap, selecting a subset of k-mers in a sequence can for many applications lead to a dramatic increase in efficiency while only losing a small amount of information. In this article, we will focus on *local* k-mer selection methods, which means that the criteria for selecting a specific k-mer should depend on the local information near the k-mer.

A popular class of local selection methods use *minimizers* (Roberts *et al.*, 2004; Schleimer *et al.*, 2003), and a lot of recent literature focuses on both practically optimizing minimizer efficiency and theoretical intrinsic properties of minimizers (Marçais *et al.*, 2018; Zheng *et al.*, 2021, 2020a,b). Recently, new non-minimizer local selection methods for k-mer selection have been proposed, including *syncmers* (Edgar, 2021) and *minimally*  *overlapping words* (Frith *et al.*, 2020).

Improvements for minimizer techniques have historically focused on optimizing for *density*, the fraction of selected k-mers. However, it often makes more sense for density to be an application dependent tunable parameter. Thus, Edgar (2021) instead propose the new metric of *conservation*, which measures the fraction of bases in a sequence which can be ‘recovered’ by k-mer matching after the sequence undergoes a random mutation process; a similar metric is also used in Frith *et al.* (2020) and Sahlin (2021a). While newer techniques have demonstrated effectiveness through empirical studies, it is not clear *why* certain methods perform well beyond heuristic notions.

*Contributions*. We make both theoretical and practical contributions in this manuscript. The first part of our article is theoretical. In Sections 2, 3 and 4, we develop a novel, more general, mathematical framework for analyzing local k-mer selection methods. We show how our framework rigorously discerns the relationship between the notion of ‘clumping’ of k-mers alluded to in Frith *et al.* (2020) and conservation (Theorem 3). We then mathematically analyze existing local k-mer selection methods, resulting in new closed-form expressions of conservation for various k-mer selection methods and a novel result on optimal parameter choice for open syncmers (Theorem 8). A summary of the concepts discussed in the theoretical portion of our article can be found in Table 1.

**Table 1.** Simplified definitions of concepts discussed in Sections 2 and 3

Term	Simplified definition
*q*-local k-mer selection method	Function which selects k-mers from a string based on windows of *q* consecutive k-mers
*r*-window guarantee	A k-mer selection method has this property if for every *r* consecutive k-mers, one is always selected
Minimizer	*w*-local k-mer selection method that selects the smallest k-mer (subject to some ordering) in windows of *w* consecutive k-mers
Word-based methods	1-local k-mer selection methods that select a k-mer if its prefix is in a specified set *W*
Open syncmer	1-local k-mer selection method that selects a k-mer if the smallest s-mer inside the k-mer is at the *t*th position (1-indexed)
Closed syncmer/charged context	1-local k-mer selection method that selects a k-mer if the smallest s-mer inside the k-mer is at the first or last position
Conservation	Percentage of bases in a long string *S* and a mutated version S′ that are covered by matching k-mers from *S* and S′
Spread	A k-mer selection method has this property if with high likelihood, the k-mers chosen are not too close together
Pr(f)	Probability vector of *f*, a vector of length *k* which is a precise measure of spread for a k-mer selection method
Pr(α(θ,k))	Vector of length *k* which measures the probability of runs of length ≥k in a sequence of 2k−1 Bernoulli trials
*UB*(*d*)	Vector which is an upper bound on Pr(f) computed using a union bound

The second part of our contributions is practical. In Section 5, we empirically calculate conservation for a wide range of methods for which we have no closed-form expression. We then modified the existing software minimap2 (Li, 2018) to use open syncmers, which show better conservation than the default minimap2 choice of minimizers. Our results show (i) conservation and alignment sensitivity are correlated and (ii) alignment sensitivity is increased after modifying minimap2. However, we also show that the k-mers selected by more conserved methods are also more repetitive, as predicted in Section 3.4, leading to higher runtime. We investigate how different parameterizations lead to runtime and alignment quality trade-offs for ONT cDNA mapping.

## 2 Preliminaries

We formally define local k-mer selection in this section. We give an original general formalism that extends the existing formalisms in Marçais *et al.* (2018). We also review existing local k-mer selection methods.

### 2.1 k-mer selection methods

Let Σ be our alphabet. We will be implicitly dealing with nucleotides (Σ={A,C,G,T}) for the rest of the article, although our results generalize without issues. For a string $S\in \Sigma^{*}$, we use the notation S[i,k] to mean the substring of length k starting at index *i*. We will assume our strings are 1-indexed.

Definition 1. *A k-mer selection method is a function f from the set of finite strings* $\Sigma^{*}$  *such that for* $S\in \Sigma^{*}$, f(S) contains tuples (x, i) where $x\in \Sigma^{k}$  *is a k-mer in S, and i is the starting position where x occurs.*

We will sometimes refer to a k-mer selection method as a selection method or just a method when the value of *k* is implied. We now define a *local* k-mer selection method.

Definition 2. *A method f is a q-local method if*
 

$$f(S)=\overset{|S|-(k+q-1)+1}{\underset{i=1}{\cup }}f(S[i,k+q-1])$$

*for every* $S\in \Sigma^{*}$  *of length* ≥k+q−1  *after an appropriate shift in the position of the k-mers.*

In other words, a q-local method is just defined on (k+q−1)-mers and then extended to arbitrary strings. The special case of *q *=* *1 implies that the method can be defined by examining all k-mers and deciding if each k-mer is selected or not. We will always assume that |S|≥q+k−1, and will focus only on local methods in this article. The main reason for doing so is that q-local methods have the following desirable property that is an easy result of the definition.

Theorem 1. *Let f be a q-local method. If two strings* S,S′  *share a region of length* k+q−1*, i.e.* S[i,k+q−1]=S′[j,k+q−1]*, then every k-mer in* f(S[i,k+q−1])=f(S′[j,k+q−1])  *is also in* f(S′)  *and f(S) (if ignoring the index of the starting position).*

Proof. Follows easily from the definition of q-locality.□

To see not all k-mer selection methods are local, it is not hard to see that the MinHash (Broder, 1998; Ondov *et al.*, 2016) sketch, which is computed by selecting a fixed number of k-mers hashing to the smallest values over the *entire* genome, is not local. Another method that is not local is selecting every *n*th k-mer occurring in a string because it depends on the global property of starting position. In the next section, we will give examples of local methods.

Our notion of a local k-mer selection method is more general than the notion of local schemes defined in Marçais *et al.* (2018). Local schemes are defined to be functions of the for*m*
 

$$f:\Sigma^{w+k-1}\to [0:w-1]$$

where [0:w−1]={0,…,w−1}. Local schemes essentially select exactly one k-mer from a w+k−1-mer by specifying the starting location for a specific k-mer. While local schemes give rise to *w*-local k-mer selection methods, not all local k-mer selection methods are local schemes because local selection methods may select 0 or more than 1 k-mer in a window. Local schemes are defined in such a way to satisfy a property called the *window guarantee*.

Definition 3. *A local k-mer selection method has the r-window guarantee property for r if* f(s)=∅  *for all* $s\in \Sigma^{k+r-1}$.

The window guarantee says that for every *r* consecutive k-mers, the local method will select at least one k-mer, guaranteeing that there will be no large gaps on the string for which no k-mer is selected. While the window guarantee is useful for many applications (Marçais *et al.*, 2019), in some applications such as alignment it is not necessary. A closely related notion to the window guarantee are universal hitting sets (UHS) (Ekim *et al.*, 2020; Orenstein *et al.*, 2017), which give rise to local methods with a window guarantee. To the best of our knowledge, current UHS implementations work within a relatively limited range of parameters. For example, PASHA (Ekim *et al.*, 2020) is not practical for *k *>* *16, and in the paper for removal (DeBlasio *et al.*, 2019), only a window guarantee of length 6 is tested. Therefore, we will not explore UHS in this article, however, research in UHS is active and we expect these limitations to be improved upon in the future.

Another important property of a selection method is the *density*.

**Fig. 1.**
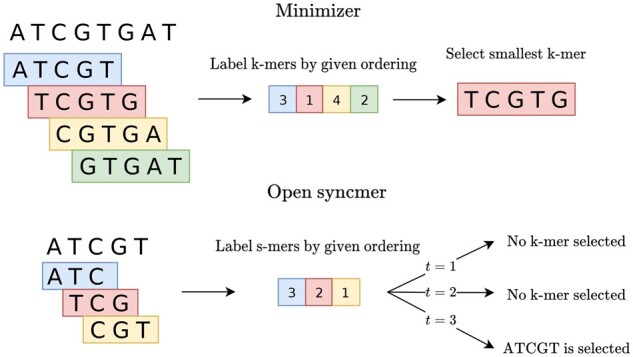
Visual example of the minimizer and open syncmer selection methods with *k *=* *5, *w *=* *4 and *s *=* *3 over some arbitrary ordering on s-mers and k-mers. Minimizers are *w*-local methods, so they operate over all k+w−1-mers and select k-mers from k+w−1-mers whereas open syncmers are 1-local methods, so they select k-mers from a single k-mer, i.e. decide whether or not a k-mer is selected. The k-mer TCGTG is selected by the minimizer because it is the smallest k-mer in the window. The k-mer ATCGT is selected by the open syncmer method if and only if *t *=* *3, since the smallest s-mer is at the third position

Definition 4. *The density of a k-mer selection method f is the expected number of selected k-mers divided by the number of total k-mers in a uniformly random string with i.i.d characters S as* |S|→∞.

Given a long uniform random string *S*, by taking indicator random variables *Y_i_* for the event that the k-mer starting at *i* is in *f*(*S*), one can easily see density is equal to $\mathrm{Pr}(Y_{i}=1)$ as long as *i* is not near the edges (the start/end) of *S*. The issue near the edges is that in Definition 2, a k-mer near the start of *S* appears in less of the S[i,k+q−1] ‘windows’ than a k-mer in the middle of the string. This is known as the edge bias problem in minimizers (Edgar, 2021).

### 2.2 Overview of specific selection methods

#### 2.2.1 Minimizers

The most well-known class of local k-mer selection methods are minimizer methods, originally appearing in Roberts *et al.* (2004); Schleimer *et al.* (2003).

Definition 5. *Given a triple* (w,k,O)  *where w, k are integers and* O  *is an ordering on the set of all k-mers, a minimizer outputs the smallest k-mer appearing in a* (w+k−1)*-mer, or equivalently a window of w consecutive k-mers.*

From now on, when we specify the smallest value in a window, ties are broken by letting the leftmost k-mer be the smallest. A minimizer gives rise to a *w*-local method with a *w*-window guarantee by examining all windows of *w* k-mers and selecting k-mers inside this window. *k* and *w* are application-dependent parameters that control for density and k-mer size, so there is one free parameter which is the ordering O.

Somewhat surprisingly, the ordering, which can be string-dependent, plays a very important part of minimizer performance. We define a *random minimizer* to be a minimizer with the ordering defined by a random permutation. Schleimer *et al.* (2003) show that the density of random minimizers is approximately $\frac{2}{w+1}$. This proof is quite elegant and insightful, so we will reproduce the proof. First, we need the definition of a *charged context*.

Definition 6 (Charged contexts). *Given parameters* (w,k,O)*, a window of consecutive w + 1 k-mers (i.e. a* (w+k)*-mer) is a charged context if the smallest k-mer is the first or last k-mer in the window.*

A very similar definition of *charging* appears in Schleimer *et al.* (2003), although they do not actually define the charged context.

Theorem 2 * (*From Schleimer *et al.*, 2003). *Assuming that no window of w + 1 k-mers contains duplicate k-mers, the density of a random minimizer is* $\frac{2}{w+1}$.

Proof. The key in the proof is to note that counting the number of selected k-mers is the same as counting the *number of times a new k-mer is selected* as we check each (w+k−1)-mer in a long random string *S*.

Mathematically, let *X_i_* be the (random) position of the k-mer selected at the window of *w* k-mers starting at *i*. By the above paragraph, the expected number of k-mers is then just 1+∑i=1|S|−w−kPr(Xi=Xi+1), where the 1 is because the first window always selects a ‘new’ k-mer. Letting |S|→∞ and dividing by |S|, the density is then just Pr(Xi=Xi+1).

The next key step is to notice that Xi=Xi+1 if and only if the smallest k-mer in the window of *w *+* *1 consecutive k-mers starting at *i* is first or last k-mer, or equivalently, if this window is a *charged context*. If the smallest k-mer is the first k-mer, then *X_i_* = *i*, but $X_{i+1}\geq i+1$, and if the smallest k-mer is the last, then $X_{i+1}=i+w$ but $X_{i}\leq i+w-1$. If the smallest k-mer is at position i<j<i+w, then $X_{i}=X_{i+1}=j$ necessarily. Assuming no duplicate k-mers in a window of length *w *+* *1, the probability that the smallest k-mer is the first or last is simply $\frac{2}{w+1}$, completing the proof. □

Of course, the assumption in the above theorem is not valid. Marçais *et al.* (2018) give a true bound on the density for a random minimizer as $\frac{2}{w+1}+o(1/w)$ under some reasonable assumptions for *w* and *k*. However, with a specific ordering, one can achieve provably better densities for the same parameters. For example, the miniception (Zheng *et al.*, 2020a) algorithm finds an ordering which gives a density upper bounded by $\frac{1.67}{w}+o(1/w)$.

#### 2.2.2 Syncmers

Syncmers were first defined in Edgar (2021) to be a class of 1-local k-mer selection methods. These methods break k-mers into a window of k−s+1 consecutive s-mers, for some parameter *s *<* k*, and select the k-mer based on some criteria involving the smallest s-mers in the window. From now on, we will assume the ordering on the s-mers is random.

We have already seen such a construction; Edgar (2021) showed that charged contexts (Definition 6) are also called *closed syncmers* in the terminology of Edgar (2021), where *k *=* s* and w=k−s. Closed syncmers can be shown to have a *k—s* window guarantee, so this is our first (and only) example of a method that is 1-local and has a window guarantee. We also analyze the *open syncmer* defined in Edgar (2021) as it was suggested to perform well with respect to conservation.

Definition 7 (Open syncmer). *Let (k, s, t) be parameters with s < k and* 1≤t≤k−s+1*. Considering a k-mer as a window of* k−s+1  *consecutive s-mers, a k-mer is selected if the smallest s-mer appears at position t in the window.*

Importantly, open syncmers *may not* have a window guarantee. Consider the string AAAAA… with *t *=* *2 for any *k*, *s*. Remembering the leftmost s-mer is the smallest by convention, the smallest s-mer always occurs at the first position so no k-mers are selected. For *t =*  *1*, (Edgar, 2021) showed that a window guarantee exists, but may be very large.

The density of open syncmers and closed syncmers are, respectively, $\frac{1}{k-s+1}$ and $\frac{2}{k-s+1}$ up to a small $o(\frac{{\left(k-s+1\right)}^{2}}{|\Sigma {|}^{s}})$ error term, following the exact same argument as Edgar (2021) and Section 2.3.1 of Zheng *et al.* (2020a) for the error term.

#### 2.2.3 Word-based methods

Another class of 1-local selection methods considers a set of words $W\subset \Sigma^{*}$ and selects a k-mer if a prefix of the k-mer lies in *W*. Frith *et al.* (2020) consider possible *W*s and find ‘good’ possible choices for *W*. The intuition is that they want the words in *W* to not overlap with each other; this way selected k-mers overlap less.

They offer a simple form for *W* by letting $W=\{x\in \Sigma^{n+1}:x=\mathrm{abbb}\ldots \}$ where a=A and *b* can be any of {*C*, *T*, *G*} for the nucleotide alphabet. We will refer to this as the (*a*, *b*, *n*)-words method. The density of this method is $\frac{1}{4}\cdot {\frac{3}{4}}^{n}$. More sophisticated choices for *W* can be constructed, but we will not analyze such methods theoretically since these *W* are found by an optimization algorithm and thus hard to analyze. We will instead test against more sophisticated *W* empirically.

## 3 Analytical framework

While we have discussed intrinsic features of selection methods such as locality, density and window guarantee, we have not yet discussed the ‘performance’ of selection methods. It turns out that evaluating the performance of a method is subtle and depends on the task at hand. In certain contexts, methods cannot be compared because some tasks such as constructing de Bruijn graphs from minimizers (Rautiainen and Marschall, 2020) or counting and binning k-mers (Marçais *et al.*, 2019) require a window guarantee while other tasks such as alignment do not; it is shown in (Edgar, 2021) that when mutations are present between two genomes, the window guarantee does not necessarily ensure better k-mer matching (see Table 6 of Edgar, 2021).

To compare methods, we will fix the density across methods and evaluate a precise notion of performance which we will define below. In previous studies on minimizers (Marçais *et al.*, 2017; 2018; Zheng *et al.*, 2020a), the focus was only on optimizing the density for a fixed window size *w*, the assumption being that a method’s performance is *only reliant on the size of the window guarantee*. This is not an unreasonable assumption for certain tasks, but it is not applicable to methods without a window guarantee.

When selecting a subset of k-mers, a good method should select k-mers that are spread apart on *S*—k-mers should not overlap or clump together (Frith *et al.*, 2020). Intuitively, close together k-mers give similar information due to more base overlaps. To formalize this, in Section 3.1, we give a new, precise notion of spread. In Section 3.3, we prove an original result detailing how this formalism relates to conservation.

### 3.1 Formalizing k-mer spread

Let $S=x_{1}x_{2}\ldots$ be a long random string of fixed length with independent and uniformly random characters over Σ. We now define a key quantity associated to *f* which we will call the probability vector of *f*.

Definition 8 (Probability vector of *f*). *Let i be a position in S which is away from the edges. Define the event* $E_{j}=\{(S[j,k],j)\in f(S)\}$  *representing whether or not a k-mer at position j is selected by f, and*
 

$$\mathrm{Pr}(f,\alpha )=\mathrm{Pr}(\overset{i+\alpha -1}{\underset{j=i}{\cup }}E_{j})$$

*the probability that some k-mer is selected from* S[i,k+α−1]*. We call* Pr(f)=[Pr(f,1),Pr(f,2),…,Pr(f,k)]  *the probability vector of f.*

This notion is well-defined because our string consists of i.i.d letters, and *f* is translation invariant along the string due to locality. As long as *i* is not near the end or beginning of *S*, the choice of *i* does not matter. Note $\mathrm{Pr}(f,1)=\mathrm{Pr}(E_{i})=\mathrm{Pr}(E_{j})$ for any *j* is the probability that a random k-mer is selected by *f*, which is the density of *f*.

Pr(f,α) is a measure of how positionally spread out the events $E_{i},\ldots ,E_{i+\alpha -1}$ are, where Pr(f,α) is maximized when all events are disjoint. The interpretation follows because if all events are disjoint, then when a k-mer is selected at position *i*, no k-mer is selected at positions i+1,…i+α−1 so the selected k-mers are spread out along the string. On the other hand, if all events only occur simultaneously, then Pr(f,α) is small and the selected k-mers are clumped together.

We can get a natural upper bound for Pr(f) because $\mathrm{Pr}(f,\alpha )=\mathrm{Pr}(\overset{i+\alpha -1}{\underset{j=1}{\cup }}E_{j})$ so the union bound give*s*
 

Pr(f)≤[min(1,d),min(1,2d),…]=UB(d)

where ≤ means over all components, remembering that $\mathrm{Pr}(E_{j})=d$ is the density. The asymptotically optimal minimizer constructed in Marçais *et al.* (2018) with density 1/w actually achieves this upper bound since Pr(f,1)=1/w and Pr(f,w)=1.

On a technical note, one can actually see that for 1-local methods, Pr(f,α) is equivalent to Pr(f(S[i,k+α−1])=∅). The issue is that for *w*-local methods, f(S[i,k+α−1]) is not defined if α<w. For example, one cannot deduce if a k-mer is selected by a minimizer method just based on the k-mer itself; a window of k-mers is needed.

### 3.2 Mutated k-mer model

Let $S\prime =x_{1}^{\prime }x_{2}^{\prime }\ldots$ be a mutated version of *S* such that $\mathrm{Pr}(x_{i}^{\prime }=x_{i})=1-\theta$ and Pr(xi′=xi)=θ, where the mutated character is uniform over the rest of the alphabet and θ∈[0,1] is some mutation parameter. A similar model for k-mer mutations is used in Blanca *et al.* (2021). We give a mathematical definition of the conservation metric from Edgar (2021).

Definition 9 (Conservation). *Given a k-mer selection method f and parameter θ, let the set of conserved bases be*
 

$$\begin{array}{c} B(f,\theta ,k)=\{i:(x,j)\in f(S)\cap f(S\prime )\;\text{for}\;\text{some}\\ \;\;\;\;\;\;\;\;\;\;\;\;\;\;\;\;\;j\;\in \;\{i-k+1,i-k+2,\ldots ,i\}\} \end{array}$$

*Define the conservation to be* $\text{Cons}(f,\theta ,k)=E[\frac{\left|B(f,\theta ,k)\right|}{\left|S\right|}].$

The set B(f,θ,k) is the set of bases for which (i) a k-mer is selected by *f* overlapping the base and (ii) this k-mer is *unmutated* from *S* to S′. In our definition, the position of matching/conserved k-mers has to be the same in *S* and S′, so we disregard spurious matches across the genome.

### 3.3 Relating conservation and spread

We now show that Cons(f,θ,k) and Pr(f), which captures k-mer spread, are related. To calculate Cons(f,θ,k), we let *X_i_* be indicator random variables where *X_i_* = 1 if i∈B(f,θ,k). By linearity of expectation $\text{Cons}(f,\theta ,k)=\sum_{i=1}^{\left|S\right|}EX_{i}/|S|.$ The *X_i_* are not independent; if i∈B(f,θ,k), then it is likely that i+1∈B(f,θ,k) as well. If i=j and both lie away from the ends of *S*, we get that $\mathrm{Pr}(X_{i}=1)=\mathrm{Pr}(X_{j}=1)$. If k≪|S|, the contribution from positions near at the edges of the string is small. Therefore, we will make the assumption $\text{Cons}(f,\theta ,k)=EX_{i}=\mathrm{Pr}(X_{i}=1)$ for some *i* in *S* away from the edges.

#### 3.3.1 Understanding mutation configurations

Given the base *i*, the k-mers covering *i* on S′ are S′[i−k+1,k],…,S′[i,k] so the substring of all covered bases is S′[i−k+1,2k−1]. We examine how mutations change the k-mers for these bases. We can consider these 2k−1 bases on S′ as 2k−1 Bernoulli trials with success probability 1−θ corresponding to an unchanged base. We call possible sequences of Bernoulli trials configurations of the mutations. A similar notion of configurations is used in Chaisson and Tesler (2012).

**Fig. 2.**
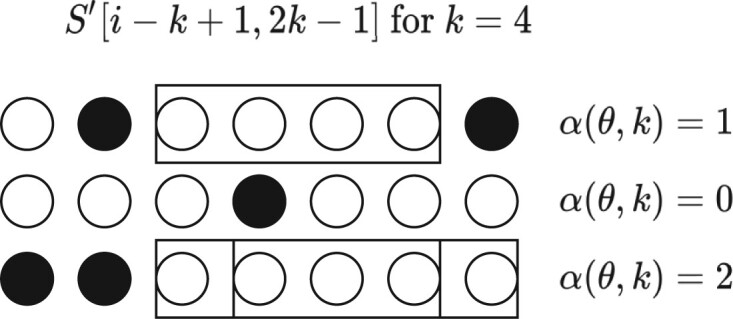
Three examples of mutation configurations. Circles represent bases of S′ around *i*, while black and white indicate mutated or unmutated bases, respectively. Boxes indicate an unmutated k-mer overlapping position *i*

Given some configuration, we will get α(θ,k) unmutated k-mers overlapping *i*, an unmutated k-mer being one for which all bases are unmutated. These unmutated k-mers are candidates for being in f(S)∩f(S′). In Figure 2, we show graphically how different configurations lead to a different value of α(θ,k).

From Figure 2, clearly α(θ,k) is a random variable corresponding to the number of successful runs of length exactly *k* in 2k−1 trials, or equivalently α(θ,k)=max{0,L(θ,k)−k+1} where L(θ,k) is the longest run in a sequence of 2k−1 Bernoulli trials with failure probability *θ*. This problem is well-studied; see Chapter 5.3 in Uspensky (1965) for a closed-form solution of general run length probabilities.

#### 3.3.2 Calculating $EX_{i}$ by conditioning

Conditioning on *α*, we get

$$EX_{i}=\sum_{a=1}^{k}\mathrm{Pr}(i\in B(f,\theta ,k)|\alpha (\theta ,k)=a)\mathrm{Pr}(\alpha (\theta ,k)=a).$$

Pr(i∈B(f,θ,k)|α(θ,k)=a) is the probability that some k-mer is selected from the *a* consecutive unmutated k-mers in *S* and S′. In the language of Definition 8, letting $E_{l}^{\prime }$ be the same event as *E_l_* but over S′,

(1) $$\mathrm{Pr}(i\in B(f,\theta ,k)|\alpha (\theta ,k)=a)=\mathrm{Pr}(\overset{a-1}{\underset{j=0}{\cup }}\left(E_{j+x}\cap E_{j+x}^{\prime }\right))$$

where *x* is arbitrary and can be thought of as the starting position of the first unmutated k-mer (the probability does not depend on *x*). Locality comes into play now; if *f* is a 1-local method, then the event $E_{j+x}$ is true if and only if $E_{j+x}^{\prime }$ is true. This follows because S′[j+x,k]=S[j+x,k] by the assumption, and by 1-locality, this k-mer is in f(S)∩f(S′) after reindexing if and only if this k-mer is selected by *f*. Hence $E_{j+x}\cap E_{j+x}^{\prime }=E_{j+x}$ for 1-local methods. Now we see that the right-hand side of Equation 1 is exactly Pr(f,α) from Definition 8, giving us Theorem 3.

Theorem 3. *Let*  Pr(α(θ,k))=[Pr(α(θ,k)=1),Pr(α(θ,k)=2),…Pr  (α(θ,k)=k)]  *and f be a 1-local method. Then*
 

(2) $$\text{Cons}(f,\theta ,k)=EX_{i}=\mathrm{Pr}(f)\cdot \mathrm{Pr}(\alpha (\theta ,k)).$$

*If f is not 1-local, then*
 

$$\text{Cons}(f,\theta ,k)=EX_{i}\leq \mathrm{Pr}(f)\cdot \mathrm{Pr}(\alpha (\theta ,k))$$

*follows from the trivial upper bound* $\mathrm{Pr}(E_{j+x})\geq \mathrm{Pr}(E_{j+x}\cap E_{j+x}^{\prime }).$

If *f* is not 1-local, then Ej+x∩Ej+x′=Ej+x. For minimizers, this is the *context dependency problem*; if a k-mer is selected in *S* and the same k-mer is also in S′, it may not be selected due to mutations in the window. Thus, 1-local methods are inherently superior for tasks that require k-mer matching for mutated strings (e.g. alignment), and the importance of this property has been recognized in other contexts as well (Ekim *et al.*, 2021).

#### 3.3.3 Calculating Pr(α(θ,k))

Even though the successful runs problem is solved for general parameters, the probability formula is derived by manipulating generating functions and is a bit unruly. For 2k−1 trials and ≥k runs, we provide a more straight-forward derivation in Supplementary Section S1, where we also show plots for Pr(α(θ,k)) over varying parameters.

Theorem 4. *For* 2k−1  *i.i.d Bernoulli trials with success probability* 1−θ  *and* 0≤β<k−1,

$$\begin{array}{c} \mathrm{Pr}(\alpha (\theta ,k)=\beta +1)=\mathrm{Pr}(\text{Longest}\;\text{run}\;\text{of}\;\text{successes}\;\text{is}\;k+\beta )\\ ={\left(2\;+\theta (k\;-\beta \;-2)(1-\theta \right)}^{k+\beta }\theta ). \end{array}$$

*For the case* β=k−1*, the probability of* 2k−1  *successes is just* ${\left(1-\theta \right)}^{2k-1}$.

### 3.4 Repetitive k-mers and locality

Given that 1-local methods are superior for conservation, which is a measure of sensitivity, it is natural to ask if such methods have an inherent precision trade-off. In the context of alignment, k-mer matching precision is related to spurious k-mer matchings caused by repeat k-mers, as such matches would force an aligner to evaluate several candidate alignments. In general, unique k-mer matches are easier to handle computationally than repeat k-mer matches. It may therefore be more advantageous for a local selection method to select non-repetitive k-mers. We show that indeed, locality is related to repetitive k-mer selection.

Theorem 5. *Let f_1_ be a 1-local method and f_n_ be an* n−*local method with n > 1 such that the densities of both methods are equal. That is, for a long uniformly random string S*, $\left|f_{n}(S)|∼|f_{1}(S)\right|$*. Then*
 

$$E[\#\text{unique k-mers}\;\text{in}\;f_{n}(S)]>E[\#\text{unique k-mers }\text{in}\;f_{1}(S)].$$

Proof. Given any local method *f*, by linearity of expectation, we have

$$E[\#\text{unique k-mers}\;\text{in}\;f(S)]=\sum_{x\in f(S)}\mathrm{Pr}(x\;\text{is}\;\text{unique}\;\text{in}\;f(S)).$$

Writing x∈U[f(S)] to mean *x* is unique in *f*(*S*) and doing the same for U[S], where we abuse notation to mean unique over all k-mers of *S*, we can condition on x∈U[S] to get

Pr(x∈U[f(S)])=Pr(x∈U[f(S)] | x∈U[S]) Pr(x∈U[S])                                 +Pr(x∈U[f(S)] | x∈U[S]) Pr(x∈U[S]).

Now notice that if x∈U[S], then x∈U[f(S)] follows. Furthermore, since *S* is uniformly random, Pr(x∈U[S]) is the same regardless of *x*. Therefore, the first term is independent of the method.

However, for 1-local methods, $x\in U[f_{1}(S)]$ can *never* be true if *x* is not unique in *S* by the definition of 1-locality. However, for an *n*-local method, it is certainly possible for $x\in U[f_{n}(S)]$ even if *x* is not unique in *S*. Hence, $\mathrm{Pr}(x\in U[f_{1}(S)])<\mathrm{Pr}(x\in U[f_{n}(S)])$. Assuming that $\left|f_{1}(S)|=|f_{n}(S)\right|$ up to a small relative error when *S* is large, the theorem follows. □

The above result show that 1-local methods may cause more repetitive matches than other methods such as minimizers. In fact, the above proof shows that *all* 1-local methods have the same expected number of unique k-mers if *S* is uniformly random. The same idea in the proof can be re-applied to show that the average expected multiplicity over all k-mers (i.e. how many times it repeats in the *f*(*S*)) is also higher when using a 1-local method. We explore the consequences of this in Section 5.2.3.

## 4 Mathematical analysis of specific methods

### 4.1 Syncmers

Let *k*, *s*, *t* be given and *f* be either a closed or open syncmer method. To calculate Pr(f,α), we need to analyze the *α* consecutive k-mers. Breaking these k-mers into s-mers, we get a window of k−s+α s-mers. Assume that all s-mers in the window are distinct. For uniform random strings, as shown in the proof of Lemma 9 in Zheng *et al.* (2020a), the probability of two identical s-mers appearing in a window is upper bounded by $\frac{{\left(k-s+\alpha \right)}^{2}}{|\Sigma {|}^{s}}$. For minimap2 (Li, 2018), the default parameters are *k *=* *15 and *w *=* *10. To achieve approximately the same density using an open syncmer, we need *s *=* *10, and it is easy to show that this probability is very small. Since all s-mers are distinct and we assume a random ordering on all s-mers, the relative ordering of s-mers in this window is a uniformly random permutation in $\sigma \in S_{k-s+\alpha }$ where σ(i) is the relative ordering of position *i* in the window. Determining whether or not a k-mer is selected then amounts to analyzing a random permutation’s smallest elements (see Supplementary Fig. S2).

Now given a permutation $\sigma \in S_{k-s+\alpha }$, we consider all windows [σ(i),σ(i+1),…,σ(i+k−s)] of size k−s+1 corresponding to the s-mers inside a k-mer starting at position *i*. The permutation is ‘successful’ if one of these k-mers is chosen by *f*. We now count the number of successful permutation for open syncmers and closed syncmers.

Theorem 6 (Successful permutations for closed syncmers). *Let* CS(α,k,s)  *be the number of permutations in* $S_{k-s+\alpha }$  *such that for some window* [σ(i),…,σ(i+k−s)]*, either* σ(i)  *or* σ(i+k−s)  *is the smallest element in the window. If* α≤k−s,

(3) CS(α,k,s)=2α(k−s+α−1)!.

*If* α>k−s*, then* CS(α,k,s)=(k−s+α)!.

Corollary 1. *If f is a closed syncmer method, then*
 

$$\mathrm{Pr}(f)=[\frac{2}{k-s+1},\frac{4}{k-s+2},\ldots ,\frac{2(k-s-1)}{2k-2s-1},1,\ldots ,1].$$

We prove this theorem in Supplementary Section S2. The corollary follows by seeing that Pr(f,α)=CS(α,k,s)/(k−s+α)!, which comes from our discussion about how random consecutive k-mers give rise to uniformly random permutations under our assumptions. Notice that Pr(f,1)=2/(k−s+1) and Pr(f,k−s)=1 which is in line with the density and window guarantee discussed in Table 2.

We can also count open syncmers. Unfortunately, the number of permutations is only determined as a recurrence relation and the formula is not as nice. Theorem 7 is proved in Supplementary Section S2.

**Table 2.** Properties of discussed methods

Method (parameters)	(*q*)-locality	Density	(*r*)-window guarantee
Random minimizer (*w*, *k*)	*w*	∼2/(w+1)	*w*
Miniception $\left(w,k,k_{0}\right)$	*w*	≤1.67/w+o(1/w)	*w*
Open syncmer (*k*, *s*, *t*)	1	∼1/(k−s+1)	$\infty^{*}$
Closed syncmer (*k*, *s*)	1	∼2/(k−s+1)	*k - s*
Words-based method (*W*)	1	depends on *W*	$\infty^{*}$
(*a*, *b*, *n*)-words method	1	= $\frac{1}{4}\cdot {\frac{3}{4}}^{n}$	∞

*Note*: The ∼ sign denotes up to a small error term. ∞ means that there is no window guarantee. Words-based methods and open syncmers may have a window guarantee for some parameters, i.e. if *t =1* for open syncmers, but usually do not.

Theorem 7 (Successful permutations for open syncmers). *Using parameters k, s, t as defined in Definition 7, let* τ=t−1  *and* OS(α,k,s,t)  *be the number of permutations in* $S_{k-s+\alpha }$  *such that for some window* [σ(i),…,σ(i+k−s)]  *the smallest element is* σ(i+τ)*. Define* $\ell_{1}=\tau ,\ell_{2}=k-s-\tau$*. Then*
 

$$OS(\alpha ,k,s,t)=\alpha (k-s+\alpha -1)!+R(\alpha ,k,s,t,\ell_{1})+R(\alpha ,k,s,t,\ell_{2}).$$

*We define* R(α,k,s,t,ℓ)  *as*
 

$$R(\alpha ,k,s,t,\ell )=\sum_{\beta =1}^{\ell }{\left(k-s+\alpha -1\right)}_{\beta -1}OS(\alpha -\beta ,k,s,t)$$

*where the subscript indicates falling factorial, and* OS(α−β,k,s,t)=0  *if* β≥α.

One can divide OS(α,k,s,t) by (k−s+α)! to get Pr(f,α) as previously discussed. Although lacking a nice closed-form, it still allows us to determine the optimal choice of parameter *t*. In Edgar (2021), the impact of parameter *t* was investigated on the performance of open syncmers, but no explicit conclusion was made about how to choose *t*. Below we prove a theorem that says that the parameter *t* should be chosen to be the middle position of a window of k−s+1 s-mers.

Theorem 8. *Let* $\hat{t}=\left\lceil \frac{k-s+1}{2}\right\rceil$*. Then* $OS(\alpha ,k,s,\hat{t})\geq OS(\alpha ,k,s,t)$  *for any valid choice of t.*

We prove Theorem 8 in Supplementary Section S2. Note that, t↦k−s+2−t gives the same Pr(f) by Theorem 7. Notice that if k−s+1 is even, then the preceding remark shows that there are two optimal values for *t*. By Theorems 3 and 8, we can rigorously justify the optimal value for *t* to maximize conservation. Thus, *t* is not actually a free parameter when optimizing for conservation.

### 4.2 Random minimizer

Let *w*, *k* be parameters for a random minimizer method *f*. To calculate Pr(f,α), as opposed to the analysis in the syncmer section where we look only at *α* consecutive k-mers, we now have to look *all* k-mers in all windows containing any of these *α* consecutive k-mers. As in the syncmer case, we will assume all k-mers are distinct.

Given *α* consecutive k-mers, we need to also know the ordering of the *w‒*1 k-mers to the left and to the right of these *α* k-mers because they are included in some window containing one of these consecutive k-mers. This gives us α+2(w−1) k-mers in total, with their relative orders corresponding to a permutation in $S_{\alpha +2(w-1)}$ (see Supplementary Fig. S4 for visual).

We proceed by counting permutations corresponding to some k-mer being chosen by a random minimizer method. We first define a function M(n,w,α,p) to count permutations in *S_n_* satisfying a general condition.

Theorem 9 (Successful permutations for random minimizers). *Given parameters* (n,w,α,p)  *with* p+α−1≤n*, let* M(n,w,α,p)  *be the number of permutations in S_n_ such that for some window* [σ(i),…,σ(i+w−1)]*, the smallest element is one of* σ(p),σ(p+1),…,σ(p+α−1)*. Then*
 

$$M(n,w,\alpha ,p)=\left\{\begin{array}{cc} \left(a)(n-1)!+\tilde{R}(n,w,\alpha ,\tilde{\ell_{1}}\right) & \text{for}\;w\leq n\\ +\tilde{R}(n,w,a,\tilde{\ell_{2}}) & \\ 0 & \text{for}\;w>n \end{array}\right\}$$

*where* $\tilde{\ell_{1}}=p-1,\;\tilde{\ell_{2}}=n-(p+\alpha -1)$  *and using* ${\left(x\right)}_{n}$  *to mean the falling factorial*,

$$\tilde{R}(n,w,\alpha ,\ell )=\sum_{\beta =1}^{\tilde{\ell }}M(n-\beta ,w,\alpha ,\tilde{\ell }-\beta +1)\cdot {\left(n-1\right)}_{\beta -1}.$$

The specific choice of parameters (n,w,α,p)=(2(w−1)+α,w,α,w) corresponds to the number of successful permutations for the minimizer given *α* consecutive k-mers since the *p* parameter describes the leftmost position of the first unmutated k-mer covering position *i*. Therefore, as before, Pr(f,α)=M(2(w−1)+α,w,α,w)/(2(w−1)+α)!. This theorem is proved in Supplementary Section S3.

It was shown to us recently (Spouge, 2022) that for the choice of parameters corresponding to the minimizer situation, this formula has a greatly simplified form of

$$1-\mathrm{Pr}(f,\alpha )=\frac{\left(w-\alpha +1)*(w-\alpha \right)}{(w+1)w}.$$

When *α *= 1, we get the desired result that the density is $\mathrm{Pr}(f,1)=\frac{2}{w+1}$ which agrees with Theorem 2. Note that, the exact equality for Theorem 3 holds only when *f* is a 1-local method, which minimizers are not. We give an example that shows how context dependency leads to lower conservation in Supplementary Section S3.1.

### 4.3 (*a*, *b*, *n*)-words method

We can also derive the probability vector for the previously (*a*, *b*, *n*)-words method which selects k-mers based on their prefix. We prove this result in Supplementary Section S4.

Theorem 10. Pr(f,α)  *under the (a, b, n)-words method is*
 

$$\sum_{i=1}^{\alpha }{\left(-1\right)}^{i+1}\frac{3^{ni}}{4^{i(n+1)}}\left(\begin{array}{c} \alpha -n(i-1)\\ i \end{array}\right)$$

*where* $\left(\begin{array}{c} x\\ y \end{array}\right)=0$  *if x < 0.*

## 5 Empirical results

We perform two sets of experiments. In Section 5.1, we compare Cons(f,θ,k) analytically and through simulations for a wider range of methods compared with previous studies (Edgar, 2021; Frith *et al.*, 2020) which focused only on simple minimizer methods and variations on their own methods. In Section 5.2, we modify the minimap2 (Li, 2018) software to use open syncmers and demonstrate that alignment sensitivity is increased, which is in agreement with our previous theory on conservation. However, despite the sensitivity increase given by open syncmers, the additional computational time as a result of increased repetitive k-mer indexing (see Section 3.4) due to 1-locality implies that optimizing for speed and time is not as simple as optimizing for conservation.

### 5.1 Comparing Cons(f,θ,k) across different methods

In Section 4, we derived Pr(f) for four methods: closed and open syncmers, minimizers and (a,b,n)−words. Since all but minimizers are 1-local methods, we can calculate Cons(f,θ,k) in closed-form for these three methods using Theorem 3. In this section, we empirically calculate Cons(f,θ,k) for three more methods mentioned in Section 2.2. Fixing some density *d*, we compare Cons(f,θ,k) to UB(d)·Pr(α(θ,k)), where *UB*(*d*) is defined in Section 3.1 as the upper bound on probability vectors. For the miniception method, it is not obvious how parameters affect *d* so we let $k_{0}=k-w$ for d=2/(w+1) as suggested in Zheng *et al.* (2020a), and then modify *k*_0_ and *w* slightly until we get the density close to *d*. For the words method, we use two choices of *W* as *W*_4_ and *W*_8_ (Supplementary Section S6), where the corresponding methods for *W*_4_ has density 1/4 and *W*_8_ has density 1/8. These sets are empirically found to perform well in Frith *et al.* (2020).

For methods with a closed-form for Cons(f,θ,k), we plot the exact value. For methods without a closed-form, we ran 100 simulations with |S|=50 000. We report mean and 95% confidence intervals (assuming normality) of Cons(f,θ,k) for these methods. Code can be found at https://github.com/bluenote-1577/local-kmer-selection-results.

**Fig. 3.**
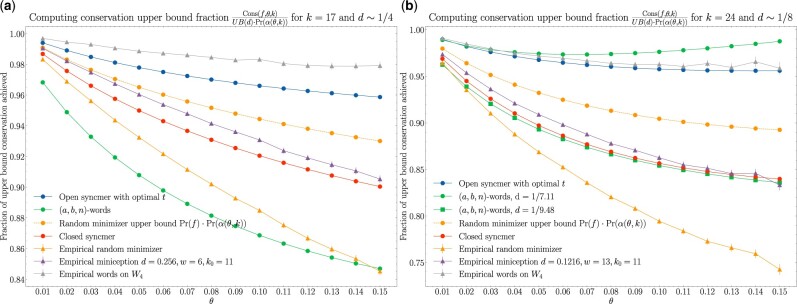
Fraction of upper bound achieved for conservation. 95% confidence intervals are built from 100 simulations for methods with empirically deduced values. Note that, some methods have different densities due to parameter constraints; this is mentioned in the labels

We first fix d=1/4,k=17 and plot the fraction of upper bound achieved, $\frac{\text{Cons}(f,\theta ,k)}{\mathrm{Pr}(\alpha (\theta ,k))\cdot UB(d)}$, for all methods over a range of *θ*. We then fix d=1/8,k=25 and do a similar plot. For the (*a*, *b*, *n*)-words method, the closest choice of parameters leading to the most similar density is *n *=* *2, 3 which gives density =9/64∼1/7.11 and 27/256∼9.48; we plot both of these for reference. Both results are shown in Figure 3.

The results in Figure 3 show that the words method based on the set *W*_4_, *W*_8_ and the open syncmer methods perform well compared with the other methods. The large drop in conservation between the empirical random minimizer and the upper bound for the random minimizer indicates that context dependency plays a highly non-trivial role in conservation. We note that for *d *=* *1/4, the (*a*, *b*, *n*)-words method performs poorly as it does not take advantage of selecting k-mers with non-overlapping prefixes *n *=* *0.

These results also suggest that the best methods already achieve ≥0.96 fraction of the possible upper bound UB(d)·Pr(α(θ,k)) for reasonable parameters and error rates, so there is not room for drastic improvement.

### 5.2 Using open syncmers in minimap2

We now investigate applications to read mapping. We modified minimap2 (Li, 2018), a state-of-the-art read aligner, so that open syncmers are used instead of minimizers. We decided on using open syncmers because although its conservation is slightly less compared with word-based methods, it allows the user to choose a range of densities without constructing a new words set *W* for each density. We note that since minimap2 was designed with minimizers in mind, we expect the benefits of switching k-mer selection methods to be dampened compared with designing an aligner with open syncmers in mind.

Minimap2 aligns reads using a seed-chain-extend procedure. Roughly speaking, this works by first applying a k-mer selection method to all reads and reference genomes. Selected k-mers on the reads are then used as *seeds* to be matched onto the selected k-mers of the reference. Colinear sets of k-mer matches are collected into *chains*, and then dynamic programming-based alignment is performed to fill gaps between chains. Our modification was to swap out the k-mer selection method, originally random minimizers, to an open syncmer method instead. Our version of minimap2 can be found at https://github.com/bluenote-1577/os-minimap2.

We operate on four sets of real and simulated publicly available long-read datasets:

1. Real microbial PacBio Sequel long-reads from PacBio, available at https://tinyurl.com/uhuwvxb8 and their corresponding assemblies.
2. Real human ONT nanopore reads from Miga *et al.* (2020) available at https://github.com/marbl/CHM13 (id: rel3; downsampled and only including reads > 1 kb in length) and the corresponding assembly CHM13.
3. Simulated RNA (cDNA) ONT long-read data from Trans-NanoSim (Hafezqorani *et al.*, 2020).
4. Simulated PacBio long-reads on human reference GRCh38 using PBSIM (Ono *et al.*, 2013).

We discuss the first three listed datasets in the following sections, and discuss the simulated PacBio experiment in Supplementary Section 7.1.

#### 5.2.1 Chaining score improvement

**Fig. 4.**
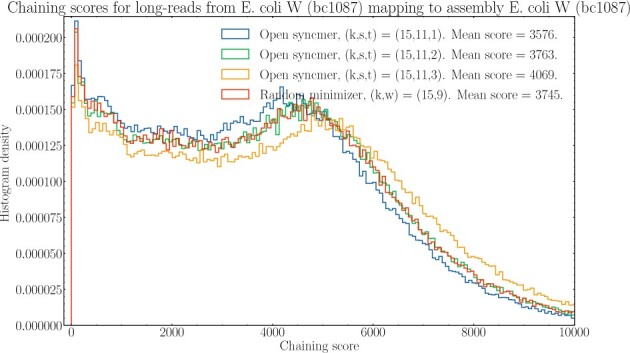
Histogram of chaining scores corresponding to alignments of reads for *E.coli* W (bc1087) in against its assembly. Minimap2 with open syncmers and minimizers were compared against each other with parameters chosen so that density is fixed at 1/5. Mean chaining scores are given in the legend. *t *=* *3 is the optimal value of *t* by Theorem 8

For each alignment of a read, minimap2 computes a *chaining score* (Section 2.1 in Li, 2018). This chaining score measures the goodness of the best possible chain for that alignment. Roughly speaking, if k-mers in a chain overlap and do not have gaps, the chaining score is high. We took the long-read sequences and assembly for *E.coli* W (bc1087) in the dataset and compared the mappings for open syncmers versus minimizers. This is shown in Figure 4.

For *t *=* *3, the optimal parameter of *t* with k=15,s=11, open syncmers leads to better chaining scores. The theoretical value of Cons(f,θ,k) increases as *t* increases for open syncmer methods because the vectors Pr(f) are increasing component-wise as *t* increases; see Supplementary Figure S3. Therefore, the increase in mean chaining score as *t* increases is suggestive that goodness of chaining is indeed correlated with conservation, even for PacBio reads which have higher rates of indels than substitutions (Dohm *et al.*, 2020). This suggests that the results from the conservation framework is still valid under realistic mutation models.

Naturally, empirical densities may deviate from theoretical densities for minimizers (Marçais *et al.*, 2017). To verify that the increase in chaining was not because of open syncmers having higher density than minimizers, we calculated the empirical density for minimizers/open syncmers on the reference genome in this experiment and found that it was 1/4.867 and 1/5.059, respectively. This shows that even though the density for open syncmers was *lower* than for minimizers, the chaining score was higher.

#### 5.2.2 Improvement in number of mapped reads and quality for real datasets

We now examine how alignment sensitivity increases when using a more conserved k-mer selection method on real datasets. We fix *t *=* *3 with the same parameters for *k*, *w*, *s* as in the previous section. For the real datasets, we analyze mapping quality and number of mapped reads. The difference between conservation of k-mer selection methods is more pronounced when the rate of mutation is higher, so we test how alignment changes as the reference genomes diverge from the reads. We test this effect by aligning the reads to varying reference genomes as well. The results are summarized in Table 3.

**Table 3.** Open syncmer versus minimizer mappings for two versions of minimap2 on long-reads. We fix parameters (k,s,t,w)=(15,11,3,9) so the density is 1/5 for both seeding methods

Long-read dataset	Reference	$\left|OS\cap \bar{M}\right|$ (mapQ)	$\left|M\cap \bar{OS}\right|$ (mapQ)	|OS|	|M|	Total no. of reads	% increase in mapped reads
*E.coli* W (bc1087)	*E.coli* W (bc1087)	312 (21.56)	102 (11.57)	194 455	194 245	196 901	0.108
*E.coli* K12 (bc1106)	*E.coli* W (bc1087)	548 (20.30)	187 (11.33)	220 459	220 098	226 906	0.164
*K.pneumoniae* (bc1074)	*E.coli* W (bc1087)	11 434 (19.80)	3679 (12.10)	143 724	135 969	251 838	5.70
Downsampled human ONT (rel3)	Human—CHM13	370 (3.53)	103 (2.52)	37 819	37 552	51 210	0.711
Downsampled human ONT (rel3)	Mouse—GRCm38	2467 (2.32)	1005 (1.90)	19 214	17 752	51 210	8.23

*Note*: *OS* is the subset of reads successfully mapped using open syncmers, and *M* similarly for minimizers. $OS\cap \bar{M}$ is the set of reads which are uniquely mapped by open syncmers, and $M\cap \bar{OS}$ are reads uniquely mapped by minimizers. The average mapQ outputted by minimap2 within the set is presented as well (Section 5.2).

These results show that the number of reads that were uniquely mapped using open syncmers is consistently greater than with minimizers. In the case of human ONT reads mapping to the mouse genome, the relative increase in number of mapped reads is around 8.2%. This effect increases as the reads and reference genomes diverge more and mapping becomes more challenging.

Since longer reads are more likely to be aligned than shorter reads, the effect of open syncmers on our metric for sensitivity, the percentage increase of mapped reads, is dependent on read length. We investigated the relationship between sensitivity increase and read lengths in Table 4. We mapped rel3 human nanopore reads onto CHM13 and stratified by read length. As expected, open syncmers increase mapping sensitivity for shorter nanopore reads disproportionately compared to very long reads.

**Table 4.** Open syncmer versus minimizer mapping statistics as a function of read length for the rel3 ONT read set mapping onto CHM13

Human ONT (rel3) read lengths	$\left|OS\cap \bar{M}\right|$ (mapQ)	$\left|M\cap \bar{OS}\right|$ (mapQ)	|OS|	|M|	Total no. of reads	% change in mapped reads
100-1000 bp	546 (4.25)	146 (2.96)	6068	5668	44 397	7.05%
1000-2000 bp	165 (3.72)	38 (1.71)	3277	3150	10 097	4.03%
2000-3000 bp	53 (3.70)	14 (5.71)	1840	1801	4151	2.16%
>3000 bp	152 (3.27)	51 (2.25)	32 706	32 605	36 968	0.31%

*Note*: *OS* is the set of mapped reads with open syncmers, *M* is the set of mapped reads with minimizers. Values in parenthesis indicate average mapping qualities calculated by minimap2.

#### 5.2.3 Simulated transcriptome analysis over varying parameters

We now analyze simulated RNA (cDNA) ONT data, where we can analyze mapping precision due to having a ground truth. We chose to analyze such data because read lengths are shorter, and in Table 4, we found that the difference in mapping sensitivity was more drastic when read lengths are shorter. We used Trans-NanoSim (Hafezqorani *et al.*, 2020) a software package for simulate cDNA ONT reads. We used the provided pre-trained ‘human_NA12878_cDNA_Bham1_guppy’ model for generating reads.

After generating a set of reads from a transcriptome, we mapped the reads back to the genome (GRCh38) using the minimap2-x splice option. For every mapping, we take the primary alignment if the mapQ > 0. We designate a mapping successful if the transcript corresponding to the read overlaps the true gene, otherwise we designate it as an error. We only analyze genes found on the primary chromosomes of GRCh38 and ignore genes found on alternate loci.

**Fig. 5.**
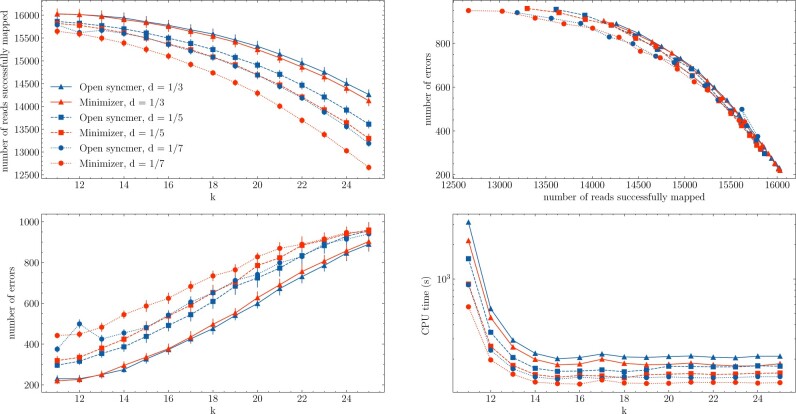
Sensitivity and precision investigation of simulated ONT cDNA data. Mapped reads were classified into success and errors based on the true transcript location. We repeated the experiment 9 times to get a 95% confidence interval. 18 104 reads were generated for each parameterization. The two left plots show that reducing k or switching to syncmers improves alignment quality. The top right plot shows the success-error curves and the bottom right plot shows the CPU time taken for each method

Based on the experiments in the previous section, it is not clear how increasing sensitivity using syncmers differs from just lowering the value of *k* to create more seed matches; this was also not investigated in (Edgar, 2021; Frith *et al.*, 2020). To investigate how each method depends on parameters *k*, *s*, *w*, we repeat the experiment over each value of *k* and over a set of fixed densities. For every parameterization, we generated 18 104 reads. The 18 104 reads are a result of simulating 20 000 reads using the Trans-NanoSim software; the software simulates garbage reads with error rates >90%, thus we only took the 18 104 reads which should be alignable. The results are shown in Figure 5. We list some of the important takeaways from this experiment below.

**Fig. 6.**
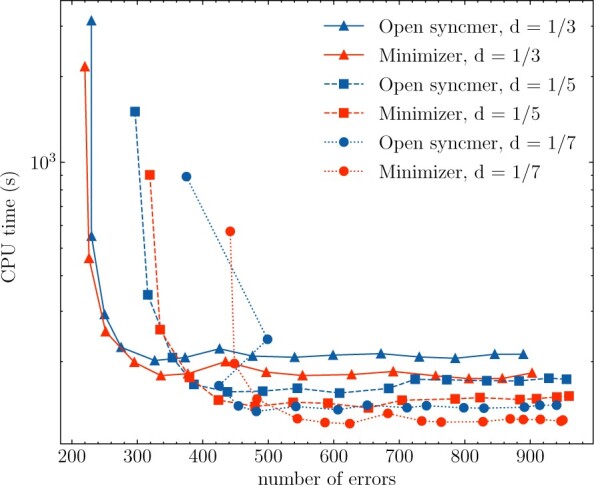
Errors versus time taken for each method. Each point is for a specific value of *k*. Syncmers provide different parameterizations when modifying density and *k*. Parameters close to the bottom-left are well-performing

1. Increasing k-mer matching sensitivity by lowering *k or* switching to open syncmers increased the quality of overall mapping (less errors *and* more successfully mapped reads).
2. Open syncmers and minimizers have similar error/success curves as seen in the top-right of Figure 5. This shows that lowering *k* and choosing more conserved methods effectively do the same thing by increasing the number of seed hits. Larger versions of this subplot are shown in Supplementary Figures S8 and S9.
3. While the quality of the mapping seems independent of lowering *k* versus using open syncmers, open syncmers allow for new parameterizations. Figure 6 shows that when considering the most optimal parameterizations (toward the bottom-left of the figure), both open syncmers and minimizers have parameters that perform well. The results suggest that 13≤k≤15 perform the best across methods, and that switching from minimizers to syncmers in this regime still gives quite optimal parameters while increasing both mapping quality and time.
4. For the case of fixed density *d* = 1/7, we noticed that syncmers may offer a sizeable error-runtime improvement for small *k*, see Supplementary Figure S10. Thus, syncmers may provide more benefit for small *d*; this can also be seen in the top-left of Figure 5. Smaller values of *d* may be necessary in cases where a large number of genomes must be indexed by k-mers and memory is an issue.
5. Syncmers take longer when *k* is fixed. For large *k*, this is primarily due to indexing speed, which we did not attempt to optimize. For smaller *k*, this seems to be due to a larger number of repetitive k-mers and spurious matches as mentioned in Section 3.4. Supplementary Figure S7 shows that the indexed set of k-mers for open syncmers contains less unique k-mers and higher k-mer multiplicity on average than minimizers.

Interestingly, we found that for even values of 1/d, the sensitivity increase for choosing open syncmers over minimizers was greatly diminished (Supplementary Fig. S6). This is perhaps related to the fact that the optimal *t* value is unique when 1/d is odd, whereas when it is even there are two equally good choices.

## 6 Conclusion

In summary, we first described a new mathematical framework for understanding k-mer selection methods in the context of conservation, which allowed us to prove results pertaining to upper bounds, optimal parameter choices and closed-form expressions for conservation. We then investigated conservation empirically and then found that augmenting minimap2 with a more conserved method increases alignment sensitivity as predicted. However, such methods may give rise to more repetitive seed matches, increasing the computational time of alignment. To optimize for quality of mapping versus speed, one should *maximize* sensitivity, which is related to quality, and *minimize* the number of repetitive k-mers in the indexed set, which is related to speed. Further research should investigate if this trade-off can be improved using new local-selection methods and how to engineer aligners that take advantage of the trade-off.

A notable result of ours is that the best methods can already achieve > 0.96 of the upper bound for conservation for certain parameters, implying that major improvements in conservation are not possible. However, real genomes do not consist of i.i.d uniform letters. There is a mix of high-complexity and low-complexity regions in genomes, so techniques for better distributed selection of k-mers should be investigated (Jain *et al.*, 2020) for example. Sequence-specific k-mer selection methods, where the selection method is specifically tuned for a certain string is another area of practical importance (Zheng et al., 2021). Theoretical problems include understanding how tight the bound given in Section 3.1 is when parameters are not in the asymptotic regime, and deeper analysis on the context dependency problem for random minimizers as well as on how locality relates to repetitiveness of selected k-mers.

An orthogonally related recent idea are strobemers (Sahlin, 2021a), which have been proposed as a k-mer alternative for sequence mapping. It has been shown that strobemers allow for much higher conservation (called match-coverage in Sahlin, 2021a) than k-mers. StrobeAlign (Sahlin, 2021b) is a new short-read aligner that combines syncmers and strobemers for extremely efficient alignment. Another example is the LCP (locally consistent parsing) technique (Hach *et al.*, 2012; Sahinalp and Vishkin, 1996), which selects varying length substrings instead of k-mers in a locally consistent manner (i.e. a version of Theorem 1 holds). Understanding selection techniques for k-mer replacements is an area of unexplored research where we believe that some of our techniques may be useful.

## Supplementary Material

btab790_Supplementary_Data
