# Supplementary material for: Theory of local k-mer selection with applications to long-read alignment
Source: Bioinformatics. 2021 Nov 19;38(20):4659–69. doi: 10.1093/bioinformatics/btab790 (PMC9563685; doi:10.1093/bioinformatics/btab790)
Supplement: btab790_Supplementary_Data [file btab790_supplementary_data.pdf]

# Appendix to Theory of local k-mer selection with applications to long-read alignment

Jim Shaw and Yun William Yu
University of Toronto

November 9, 2021

## Contents

**S1 $\Pr(\alpha(\theta, k))$ formula and figure** 1

**S2 Syncmer proofs** 2

**S3 Random minimizer probability vector** 5
S3.1 Context dependency . . . . . . . . . . 7

**S4 Proof of $(a, b, m)$-words method probability vector** 7

**S5 Comparing $\Pr(f)$** 8

**S6 Defining $W_4, W_8$** 8

**S7 Minimap2 supplementary results with open syncmers** 9
S7.1 Simulated PacBio Experiment . . . . . . . . . . 9

## S1 $\Pr(\alpha(\theta, k))$ formula and figure

**Theorem S1.1.** *For $2k-1$ i.i.d Bernoulli trials with success probabillity $1-\theta$ and $0 \leq \beta \leq k-1$,*

$$\Pr(\alpha(\theta, k) = \beta + 1) = \Pr(\textit{Longest run of successes is } k + \beta)$$
$$= \Big(2 + (k - \beta - 2)\theta\Big)\Big((1-\theta)^{k+\beta}\theta\Big).$$

*For $\beta = k-1$, the probability of $2k-1$ successes is just $(1-\theta)^{2k-1}$.*

*Proof.* Suppose $\beta < k-1$. If the maximum successful run is of length $k+\beta$ in $2k-1$ trials, this must be the *only run* of $k+\beta$ successes in a row. Label the start and end of this sequence by positions $i, j \in \{1, ..., 2k-1\}$, where $j = i + k + \beta - 1$. The possible positions of $i$ are $i \in \{1, ...., k-\beta\}$. We calculate $\sum_{\ell=1}^{k-\beta} \Pr(k + \beta \text{ sucesses in a row}, i = \ell)$.

Case 1: if $i = 1$ or $i = k - \beta$, then trial $j+1$ or $i-1$ has to be a failure respectively, otherwise the run is longer than $k+\beta$. The remaining trials can be successes or failures; it doesn't matter. Thus $\Pr(k+\beta \text{ successes in a row}, i = \ell) = (1-\theta)^{k+\beta}\theta$ for $\ell = 1$ or $k - \beta$. This contributes a $2 \cdot (1-\theta)^{k+\beta}\theta$ term to the sum.

Case 2: if $i \neq 1$ and $i \neq k - \beta$, then both of the trials $i-1$ and $j+1$ have to be failures. Again, the remaining trials can take on any value. Thus $\Pr(k+\beta \text{ successes in a row}, i = \ell) = (1-\theta)^{k+\beta}\theta^2$ for $1 < \ell < k - \beta$. There are $(k - \beta - 2)$ such terms in the sum.

Summing the probabilities together yields the result when $\beta < k - 1$. If $\beta = k - 1$ then clearly the probability is just $(1-\theta)^{2k-1}$. □

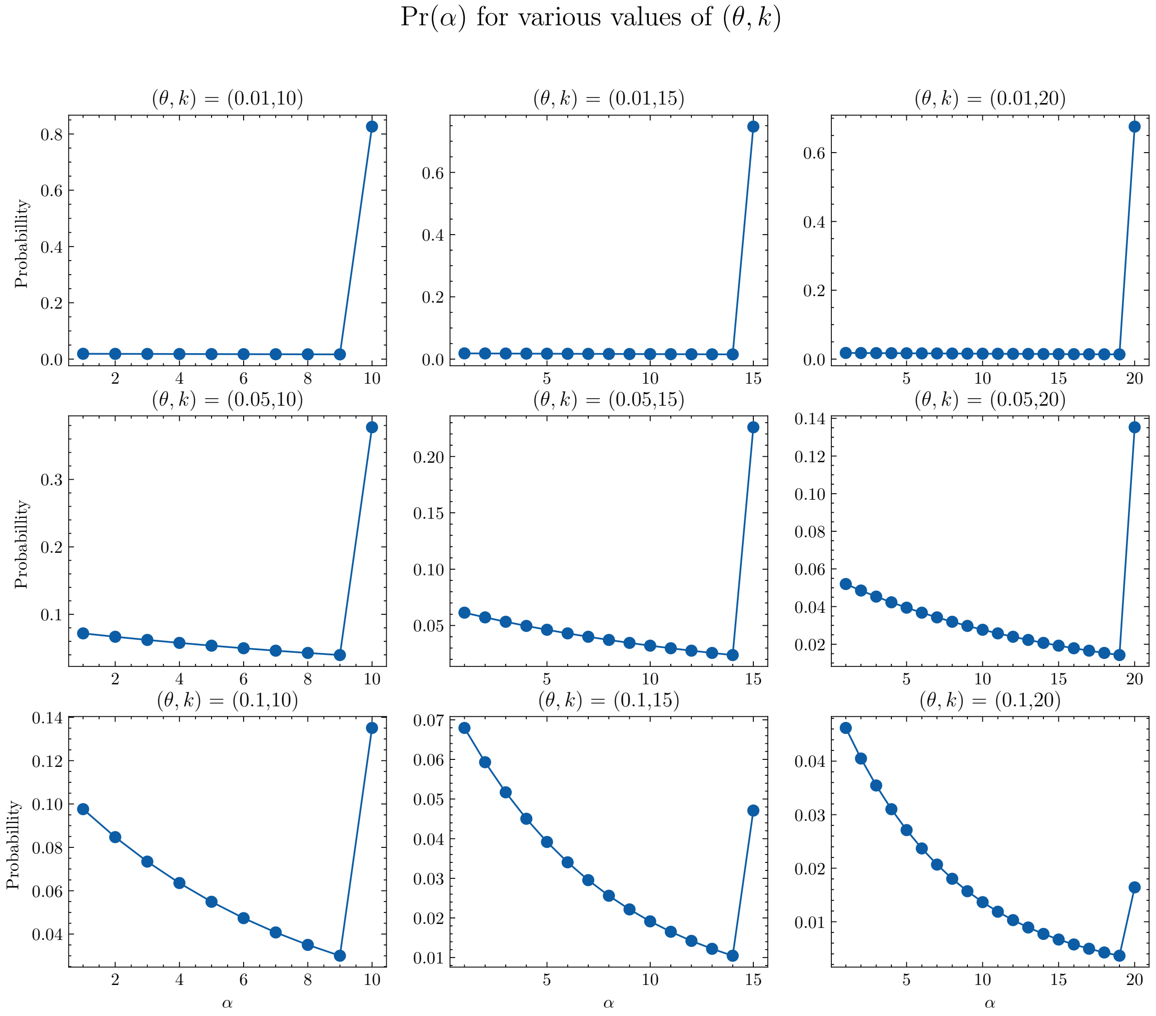


Figure 1: $\Pr(\alpha(\theta,k)) = [\Pr(\alpha(\theta,k)=1), ..., \Pr(\alpha(\theta,k)=k)]$ for various values of $\theta$ and $k$.

# S2 Syncmer proofs

**Theorem S2.1** (Successful permutations for closed syncmers)**.** *Let $CS(\alpha, k, s)$ be the number of permutations in $S_{k-s+\alpha}$ such that for some window $[\sigma(i), ..., \sigma(i+k-s)]$, either $\sigma(i)$ or $\sigma(i+k-s)$ is the smallest element in the window. If $\alpha \leq k - s$,*

$$CS(\alpha, k, s) = 2\alpha(k - s + \alpha - 1)!. \tag{1}$$

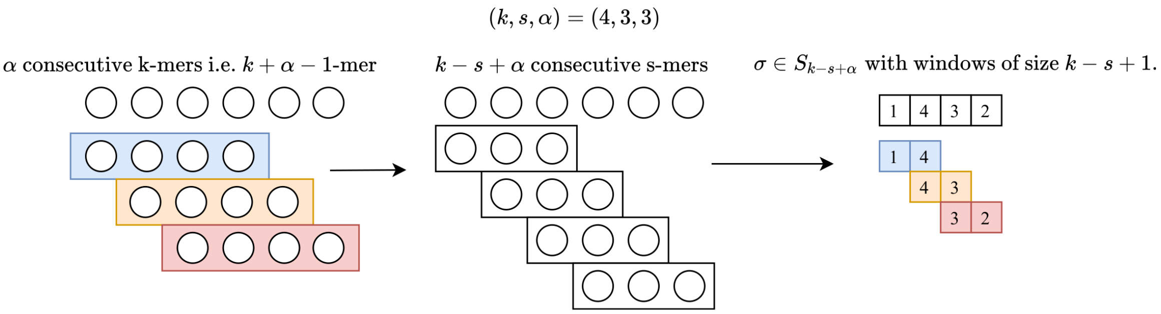


Figure 2: Graphic of how $\alpha$ consecutive k-mers give rise to a permutation in $S_{k-s+\alpha}$ where the first s-mer is the smallest, the second is the largest, etc. Colors show how k-mers correspond to windows in the permutation.

*If $\alpha > k - s$, then $CS(\alpha, k, s) = (k - s + \alpha)!$.*

*Proof.* Let $\sigma \in S_{k-s+\alpha}$. Note $\alpha$ is the number of windows in $\sigma$ of size $k-s+1$. For $i \in A_\alpha = \{1, ..., \alpha\}$, if $\sigma(i) = 1$ then the first s-mer in some window starting at $i$ is the smallest, so this permutation is successful. Otherwise, for $i \in B_\alpha = \{k - s + 1, ..., k - s + \alpha\}$ if $\sigma(i) = 1$ then the last s-mer in some window is the smallest, so it is successful.

Suppose $\sigma(i) \neq 1$ for all $i \in B_\alpha \cup A_\alpha$, so $\sigma(i) = 1$ for some $i \in \{\alpha + 1, ..., k - s\}$. Every single window contains all positions $\{\alpha + 1, ..., k - s\}$, but these positions are neither the start nor end of the window. Therefore $\sigma$ is successful if and only if $\sigma(i) = 1$ for some $i \in B_\alpha \cup A_\alpha$. Counting permutations gives $2\alpha(k - s + \alpha - 1)!$ possible permutations. □

**Theorem S2.2** (Successful permutations for open syncmers)**.** *Using parameters $k, s, t$ as defined in the definition of open syncmers let $\tau = t - 1$ and $OS(\alpha, k, s, t)$ be the number of permutations in $S_{k-s+\alpha}$ such that for some window $[\sigma(i), ..., \sigma(i + k - s)]$ the smallest element is $\sigma(i + \tau)$. Define $\ell_1 = \tau, \ell_2 = k - s - \tau$. Then*

$$OS(\alpha, k, s, t) = \alpha(k - s + \alpha - 1)! + R(\alpha, k, s, t, \ell_1) + R(\alpha, k, s, t, \ell_2).$$

*We define $R(\alpha, k, s, t, \ell)$ as*

$$R(\alpha, k, s, t, \ell) = \sum_{\beta=1}^{\ell} (k - s + \alpha - 1)_{\beta-1} OS(\alpha - \beta, k, s, t)$$

*where the subscript indicates falling factorial, and $OS(\alpha - \beta, k, s, t) = 0$ if $\beta \geq \alpha$.*

*Proof.* We condition on the position of the smallest element, i.e. the index $\beta$ for which $\sigma(\beta) = 1$. Let the set $A_\tau = \{\tau + 1, \tau + 2, ..., \tau + \alpha\}$

**(Case 1 - if $\beta \in A_\tau$).** In this case, the window

$$[\sigma(\beta - \tau), ..., \sigma(\beta - \tau + (k - s))]$$

is valid and has the desired property that $\sigma((\beta + -\tau) + (\tau)) = \sigma(\beta)$ is the smallest integer in the window, so these permutations all satisfy condition 2 above. There are $\alpha(k - s + \alpha - 1)!$ such permutations.

**(Case 2 - if $\beta < \tau+1$).** In this case, $\beta$ is left of position $t$. Notice that for all windows containing position $\beta$ will never be successful since the first window contains $\beta$ at position $< \tau + 1$, and the relative position of $\beta$ in subsequent windows will be $< \tau + 1$ as well.

The remaining windows which may still satisfy condition 2 lie are sub-windows of $[\sigma(\beta+1)...\sigma(k-s+\alpha)]$, which may be considered a permutation in $S_{k-s+\alpha-\beta}$ after relabelling elements to be in $\{1, ..., k - s + \alpha - \beta\}$ to preserve the relative order.

This new permutation has to satisfy condition 2, and the number of such permutations is exactly $OS(\alpha - \beta, k, s, t)$. We have to multiply by an additional $(k - s + \alpha - 1)_{\beta-1}$ to count the possible values for the $\beta - 1$ entries to the left of $\beta$, each of which give the same permutation in $S_{w+a-b}$ after relabelling. Summing over $b = 1, ..., \tau = \ell_1$ gives the $R(\alpha, k, s, t, \ell_1)$ term.

**(Case 3 - if $\beta > \tau + \alpha$).** This case is identical to case 2 and the same argument works after flipping directions. This works by summing over the $\ell_2 = k - s - \tau$ possible positions $\beta \in \{k - s + \alpha, k - s + \alpha - 1, ..., \tau + 1 + \alpha\}$ and using a the same relabelling after cutting off a portion of the permutation. The number of permutations for $\beta = k - s + \alpha - i$ is the same as for $\beta = i$ by symmetry. Using this correspondence gives the $R(\alpha, k, s, t, \ell_2)$ term and completes the proof.

□

We now prove the following theorem.

**Theorem S2.3.** *Let $\hat{t} = \lceil \frac{k-s+1}{2} \rceil$. Then $OS(\alpha, k, s, \hat{t}) \geq OS(\alpha, k, s, t)$ for any valid choice of $t$.*

**Lemma S2.4.** *Fix $k, s, t, \alpha$ and define $(k - s + \alpha - \beta - 1)_{\beta-1} OS(\alpha - \beta, k, s, t) = \overline{OS}(\alpha, \beta, t)$. If $\gamma \geq \beta$, for any $t$, we have*

$$\overline{OS}(\alpha, \beta, t) \geq \overline{OS}(\alpha, \gamma, t).$$

*Proof of Lemma.* We show $\overline{OS}(\alpha, \beta - 1, t) \geq \overline{OS}(\alpha, \beta, t)$ for any $\beta$, which implies the result. This is equivalent to showing that

$$OS(\alpha - \beta + 1, k, s, t) \geq \frac{(k - s + \alpha - 1)_{\beta-1}}{(k - s + \alpha - 1)_{\beta-2}} OS(\alpha - \beta, k, s, t)$$

$$= (k - s + \alpha - \beta + 1) OS(\alpha - \beta, k, s, t).$$

Notice that

$$OS(\alpha - \beta + 1, k, s, t)/(k - s + \alpha - \beta + 1)! = \Pr(f, \alpha - \beta + 1)$$

and

$$OS(\alpha - \beta, k, s, t)/(k - s + \alpha - \beta)! = \Pr(f, \alpha - \beta)$$

when $f$ is an open syncmer method with fixed parameters $k, s, t$ from our correspondence between random permutations and the event a k-mer is selected by $f$. By definition, $\Pr(f, \alpha - \beta + 1) \geq \Pr(f, \alpha - \beta)$. Technically, the correspondence is only true up to a small error due to the chance of repeated k-mers appearing in a window, but one can make $OS(x, k, s, t)$ arbitrarily close to $\Pr(f, x)$ by letting the alphabet be very large, making repeats unlikely (see the Section 2.3.1 in [1]). Then

$$OS(\alpha - \beta + 1, k, s, t) \geq (k - s + \alpha - \beta + 1) OS(\alpha - \beta, k, s, t)$$

follows from $\Pr(f, \alpha - \beta + 1) \geq \Pr(f, \alpha - \beta)$, and we're done.

□

*Proof of Theorem S2.3.* We use the similar notation as Lemma S2.4 for $\overline{OS}$.

Observe that

$$OS(\alpha, k, s, t) = OS(\alpha, k, s, k - s + 2 - t)$$

since this just swaps the $\ell_1, \ell_2$ in the definition. Since $k-s+2-\hat{t} = \hat{t}$ or $\hat{t}+1$ depending on if $k-s+1$ is odd or even, we only need to prove that this inequality holds for $t < \hat{t}$. We will assume $k-s+1$ is odd for exposition since the indices are easier to handle, but the result holds either way after a slight modification.

We proceed by induction on $\alpha$ for $OS(\alpha, k, s, t)$. For the base case $\alpha = 1$, notice that clearly $OS(1, k, s, t) = OS(1, k, s, \hat{t}) = (k-s)!$. Let $\hat{\tau} = \hat{t}-1$ and $\tau = t-1$. Therefore we want the following term to be positive:

$$\begin{aligned}
OS(\alpha, k, s, \hat{t}) - OS(\alpha, k, s, t) &= \sum_{\beta=1}^{\tau} [\overline{OS}(\alpha, \beta, \hat{t}) - \overline{OS}(\alpha, \beta, t)] \\
&+ \sum_{\beta=1}^{k-s-\hat{\tau}} [\overline{OS}(\alpha, \beta, \hat{t}) - \overline{OS}(\alpha, \beta, t)] \\
&+ \sum_{\beta=\tau+1}^{\hat{\tau}} \overline{OS}(\alpha, \beta, \hat{t}) - \sum_{\beta=k-s-\hat{\tau}+1}^{k-s-\tau} \overline{OS}(\alpha, \beta, t)
\end{aligned} \tag{2}$$

By the induction assumption the first two sums are $\geq 0$ since $OS(\alpha-\beta, k, s, \hat{t}) \geq OS(\alpha-\beta, k, s, t)$ for $\beta \geq 1$. For the last term, $k-s+1$ odd gives us $k-s-\hat{\tau} = \hat{\tau}$. We can rewrite the last line as

$$\sum_{j=1}^{\hat{\tau}-\tau} \overline{OS}(\alpha, (\hat{\tau}-j+1), \hat{t}) - \overline{OS}(\alpha, (\hat{\tau}+j), t).$$

By the induction assumption, $\overline{OS}(\alpha, (\hat{\tau}-j+1), \hat{t}) \geq \overline{OS}(\alpha, (\hat{\tau}-j+1), t)$ and using Lemma S2.4 finishes the proof because $\hat{\tau}-j+1 \leq \hat{\tau}+j$ for all $j \geq 1$.

□

We show in Figure 3 what $\Pr(f)$ looks like for syncmer methods over a range of $t$.

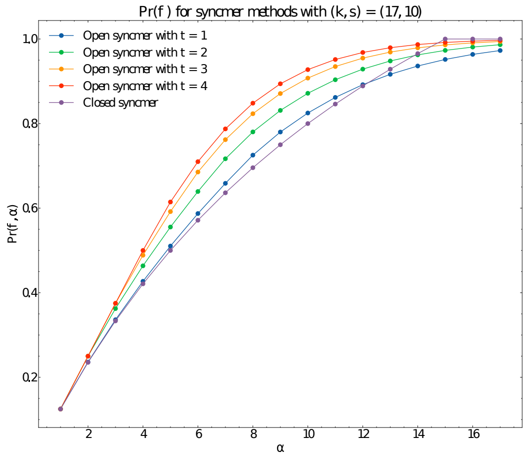


Figure 3: The probability vector for open syncmers with varying $t$ parameters $(k, s) = (17, 10)$. The $s$ parameter for the closed syncmer was chosen so that the densities are equal. We only evaluate for $t \leq 4$ because $t \mapsto k-s+2-t$ gives the same probabilities for open syncmers by Theorem S2.2.

## S3 Random minimizer probability vector

**Theorem S3.1** (Successful permutations for random minimizers)**.** *Given parameters $(n, w, \alpha, p)$ with $p+\alpha-1 \leq n$, let $M(n, w, \alpha, p)$ be the number of permutations in $S_n$ such that for some window*

$[\sigma(i), ..., \sigma(i+w-1)]$, *the smallest element is one of* $\sigma(p), \sigma(p+1), ..., \sigma(p+\alpha-1)$. *Then*

$$M(n, w, \alpha, p) = \left\{ \begin{array}{ll} (a)(n-1)! + \tilde{R}(n, w, \alpha, \tilde{\ell_1}) + \tilde{R}(n, w, a, \tilde{\ell_2}) & \text{for } w \leq n \\ 0 & \text{for } w > n \end{array} \right\}$$

*where* $\tilde{\ell_1} = p-1$, $\tilde{\ell_2} = n - (p+\alpha-1)$ *and using* $(x)_n$ *to mean the falling factorial,*

$$\tilde{R}(n, w, \alpha, \ell) = \sum_{\beta=1}^{\tilde{\ell}} M(n-\beta, w, \alpha, \tilde{\ell} - \beta + 1) \cdot (n-1)_{\beta-1}.$$

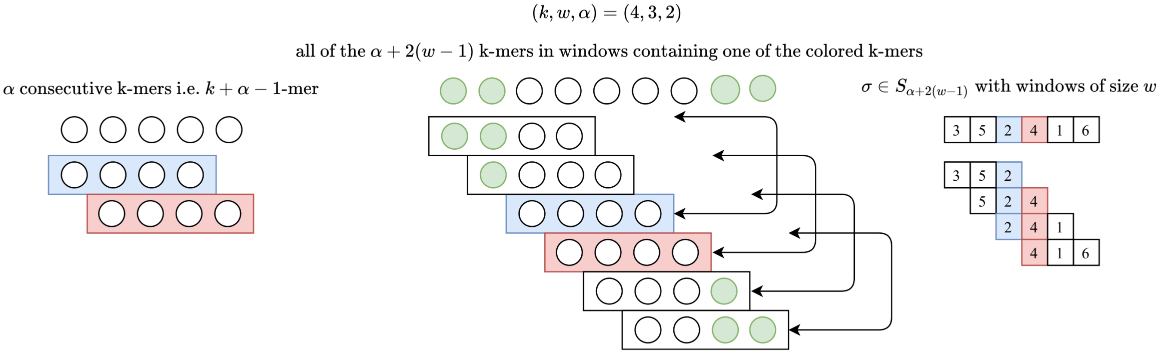


Figure 4: Graphic of how $\alpha$ consecutive k-mers gives rise to a permutation in $S_{\alpha+2(w-1)}$. The green bases correspond to extraneous bases, the colored k-mers correspond to the original $\alpha$ k-mers, and the colors/numbers in the permutation correspond to the k-mer and its relative order.

*Proof.* As in the proof of Theorem S2.2, we condition on the position of the smallest element, i.e. the index $\beta$ for which $\sigma(\beta) = 1$. Let the set $A_p = \{p, p+1, ..., p+\alpha-1\}$.

**(Case 1 - if $\beta \in A_p$).** This permutation clearly is successful. There are $\alpha(n-1)!$ such permutations.

**(Case 2 - if $\beta < p$).** In this case, $\beta$ is left of position $p$. All windows containing position $\beta$ will never be successful since $\beta \notin A_p$ and $\sigma(\beta)$ is the smallest element in the window. The only possible successful windows are the sub-windows of $[\sigma(\beta+1)...\sigma(n)]$. We can relabel the positions after shifting by $\beta$ and consider this as a new permutation on $1, ..., n-\beta$ after relabelling $\sigma(i)$ while preserving relative order. This sub-problem is exactly counted by $(n-1)_{\beta-1}M(n-\beta, w, \alpha, p-\beta)$ after multiplying by the $(n-1)_{\beta-1}$ possible values for $\sigma(i)$, $i < \beta$. Notice that if $n - \beta < w$, then there are no windows that satisfy our requirement, so $M(n-\beta, w, a, p-\beta) = 0$. Summing over $\beta < p$ gives

$$\sum_{\beta=1}^{p-1} (n-1)_{\beta-1} M(n-\beta, w, \alpha, p-\beta).$$

**(Case 3 - if $\beta > p+\alpha-1$).** The exactly same argument follows as in case 2. We see that successful windows must be sub-windows of $[\sigma(1), ..., \sigma(\beta-1)]$, so this is almost counted by $(n-1)_{n-\beta}M(\beta-1, w, \alpha, p)$ over all $\beta > p+\alpha-1$. We can shift indices to get

$$\sum_{\beta=p+\alpha}^{n} (n-1)_{n-\beta} M(\beta-1, w, \alpha, p) = \sum_{\beta=1}^{n-(p+\alpha-1)} M(n-\beta, w, \alpha, p)(n-1)_{\beta-1}.$$

To rewrite the equation to be in a similar form to case 2, one can see that $M(n-\beta, w, \alpha, p) = M(n-\beta, w, \alpha, (n-\beta)-(p+\alpha-1)+1)$ which corresponds to "flipping' the permutation on $S_{n-\beta}$ so that position $i \mapsto n-\beta-i+1$. This completes the proof. $\square$

### S3.1 Context dependency

Recall the exact expression using for calculating $\text{Cons}(f, \theta, k)$. Consider the case

$$\Pr(i \in B(f, \theta, k) \mid \alpha(\theta, k) = 1) = \Pr(E_x \cap E'_x)$$

for $w = 2$ when $f$ is a random minimizer. For $E_x$, we must consider the $2(w-1)+\alpha = 3$ relevant k-mers on $S$; call these k-mers $x_1, x_2, x_3$, where $x_2$ is the unmutated k-mer overlapping position $i$. We can see that $\Pr(E_x) = 2/3$ either from recognizing that this is the density $2/(w+1)$ or from realizing that this is equivalent to permutations in $S_3$ for which $\sigma(2) = 1$ or 2, where $\sigma(2)$ is the relative ordering of $x_2$.

However, the only k-mer on $S'$ which is guaranteed to be unmutated is $x_2$, since $\alpha = 1$. The surrounding k-mers $x'_1, x'_3$ may not be equal to $x_1, x_3$. If they are not, then

$$\Pr(E_x \cap E'_x \mid x_1 \neq x'_1, x_3 \neq x'_3) = 2/5 + (4! - 8)/5! = 8/15 < 2/3$$

by considering a random ordering on $x_1, x_2, x_3, x'_1, x'_3$ and seeing that if $x_2$ is the smallest or second smallest, it is selected on both strings, but if it is the third smallest then there are 8 permutations for which $x_2$ is not selected on one of the strings. Although $\Pr(\bigcup_{j=0}^{a} E_{j+x} \cap E'_{j+x})$ can be similarly phrased in terms of permutations, a trickier combinatorial problem arises.

## S4 Proof of $(a, b, m)$-words method probability vector

**Theorem S4.1.** $\Pr(f, \alpha)$ *under the* $(a, b, n)$*-words method is*

$$\sum_{i=1}^{\alpha} (-1)^{i+1} \frac{3^{ni}}{4^{i(n+1)}} \binom{\alpha - n(i-1)}{i}$$

*where* $\binom{x}{y} = 0$ *if* $x < 0$.

We first prove an intermediate combinatorial lemma.

**Lemma S4.2.** *Given a set of of* $\alpha$ *elements labelled* $\{1, ..., \alpha\}$*, the number of ways* $c(n+1, i)$ *to choose* $i$ *elements* $x_1, ..., x_i$ *where we order* $x_j < x_{j+1}$ *for* $j = 1, ..., i-1$ *and* $|x_j - x_{j+1}| \geq (n+1)$ *for all* $j$ *is*

$$\binom{\alpha - n(i-1)}{i}.$$

*Proof.* Let $y_0 = x_1 - 1$, $y_1 = x_2 - x_1 - 1, ..., y_i = \alpha - x_i$. The $y_i$s represent the gaps between $x_i$s and also the endpoints. A valid choice of $x_i$s corresponds exactly to a choice of $y_i$s such that each $y_i \geq n$ for $i = 1, ..., i-1$ and $y_0, y_i \geq 0$. Furthermore,

$$\sum_{j=0}^{i} y_i = \sum_{j=1}^{i-1} (x_{i+1} - x_i - 1) + \alpha - x_i + x_1 - 1 = \alpha - i$$

We can take $z_j = y_j - n$ for $i = 1, ...i-1$ and $z_j = y_j$ otherwise to get the equivalent problem of finding $z_j$ all $\geq 0$ such that

$$\sum_{j=0}^{i} z_j = \alpha - i - (i-1)n.$$

This problem is equivalent to putting $\alpha - i - (i-1)n$ indistinct balls into $i+1$ distinct jars represented by the variables $z_j$. The solution is

$$\binom{[i+1] + [\alpha - i - (i-1)n] - 1}{\alpha - i - (i-1)n} = \binom{\alpha - n(i-1)}{i}$$

as desired.

□

*Proof of Theorem S4.1.* The probability that at least one of the k-mers is selected is

$$\Pr(\bigcup_{i=1}^{\alpha} E_i)$$

where $E_i$ is the event that the $i-$th k-mer is selected. By inclusion-exclusion, we get

$$\Pr(\bigcup_{i=1}^{\alpha} E_i) = \sum_{I \subset \{1,\dots,\alpha\}} (-1)^{|I|+1} \Pr(E_I) = \sum_{i=1}^{\alpha} (-1)^{i+1} \sum_{I \subset \{1,\dots,\alpha\}, |I|=i} \Pr(E_I)$$

where $E_I = \bigcup_{i \in I} E_i$. Now note that the probability that $E_\alpha \cap E_\beta$ for $|\alpha - \beta| \leq n$ occurs is 0; k-mers with prefix *abbb...* may not be within distance $n$ from each other. If the $i$ k-mers are all distance $\geq n+1$ apart, then the probability of that event occurring is just $(\frac{3^n}{4 \cdot 4^n})^i$ because this is just the sequence *abbb...* appearing $i$ times in a string of i.i.d random letters. Therefore, denoting $c(i, n+1)$ to be the number of ways to select $i$ elements from $\{1, ..., \alpha\}$ such that each element is at least pairwise distance $n+1$ apart, we get

$$\sum_{i=1}^{\alpha} (-1)^{i+1} \sum_{I \subset \{1,\dots,\alpha\}, |I|=i} \Pr(E_I) = \sum_{i=1}^{\alpha} (-1)^{i+1} c(n+1, i) \frac{3^{ni}}{(4^{n+1})^i}.$$

Plugging in the above lemma finishes the proof.

□

# S5 Comparing $\Pr(f)$

In Figure 5, we plot all $\Pr(f)$ and $UB(d)$ where all methods have density $d = 1/7$ except for the words method, which has density $9/64 \sim 1/7.11$. This is due to the limited range of parameters choices for the methods.

# S6 Defining $W_4, W_8$

We take the words set $W_4$ as

$$\begin{aligned} W_4 = \{&rrrrry, rryrry, rryryy, ryrrrr, \\ &ryrrry, ryryry, ryyrrr, ryyrry, \\ &ryyryr, ryyryy, ryyyry, ryyyyy, \\ &ryrrry, ryryry, ryyrrr, ryyrry\}. \end{aligned} \tag{3}$$

Here $r = \{A, G\}$ and $y = \{C, T\}$ and we mean *rryrry* to be all 6-mers that satisfy this condition. This set leads to a $d = 1/4$ method, and was found by an optimization algorithm [2].

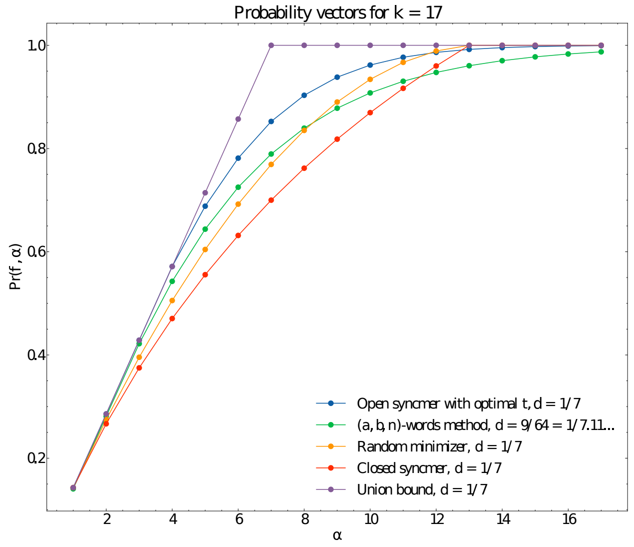


Figure 5: Comparison of $\Pr(f)$ for all methods with exact distributions derived. Note that the density for the words method is slightly smaller.

We take the words set $W_8$ as

$$\begin{aligned} W_8 = \{&rrrrrrry, rryrrryy, ryrrrryr, ryrrrryy, \\ &ryrrryry, yrrrrrry, yrrrrryr, yrrrrryy, \\ &yrrryrry, yrryrryr, yrryrryy, yryrrryy, \\ &yryrryry, yryrryyr, yryrryyy, yryryryy, \\ &yyrrrryr, yyrrrryy, yyrrryry, yyrrryyr, \\ &yyrrryyy, yyrryryr, yyrryryy, yyrryyry, \\ &yyrryyyr, yyrryyyy, yyryryyr, yyryryyy, \\ &yyryyryy, yyyryyyr, yyyryyyy, yyyyyyyr\} \end{aligned} \tag{4}$$

Here $r = \{A, G\}$ and $y = \{C, T\}$ and we mean $rryrry$ to be all 6-mers that satisfy this condition. This set leads to a $d = 1/8$ method, and was found by an optimization algorithm [2].

# S7 Minimap2 supplementary results with open syncmers

## S7.1 Simulated PacBio Experiment

For the simulated reads, we used PBSIM [3] to simulate PacBio CLR reads at 0.5 coverage across GRCh38 with mean length 15kb and 10% error rate. We report the mapping accuracy using paftools from [4]; a similar evaluation procedure is used in [5]. paftools calls an alignment correct if the overlap between an alignment and the true position of the simulated read is greater than a 0.1 fraction of all bases covering the true and mapped positions. We report accuracy and computational details in Table 1.

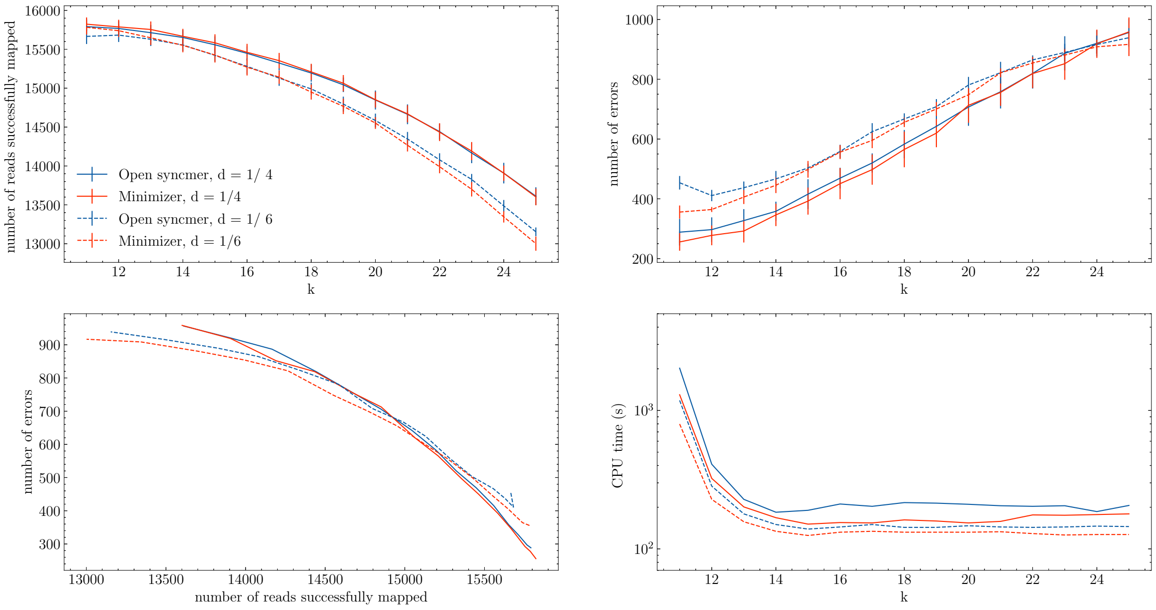


Figure 6: Sensitivity and precision investigation of simulated ONT cDNA data. Mapped reads were classified into success and errors based on the true transcript location. We repeated the experiment four times to get an 80% confidence interval. 18104 reads we used for each experiment.

| Selection Method | % Incorrect map | Indexing time (s) | Mapping time (s) | % unique selected | Density |
|---|---|---|---|---|---|
| Open syncmer | 2.96% | 109 | 1150 | 27.9% | 0.1867 |
| Random minimizer | 2.89% | 95 | 979 | 37.0% | 0.1967 |

Table 1: Alignment of PBSIM simulated PacBio reads on human genome GRCh38 with 103630 reads, mean length 15kb, and 10% error. $(k, s, t, w) = (15, 11, 3, 9)$. Number of unmapped reads was negligible. % of unique selected k-mers are k-mers selected by syncmers/minimizers from the reference that are unique over all indexed k-mers.

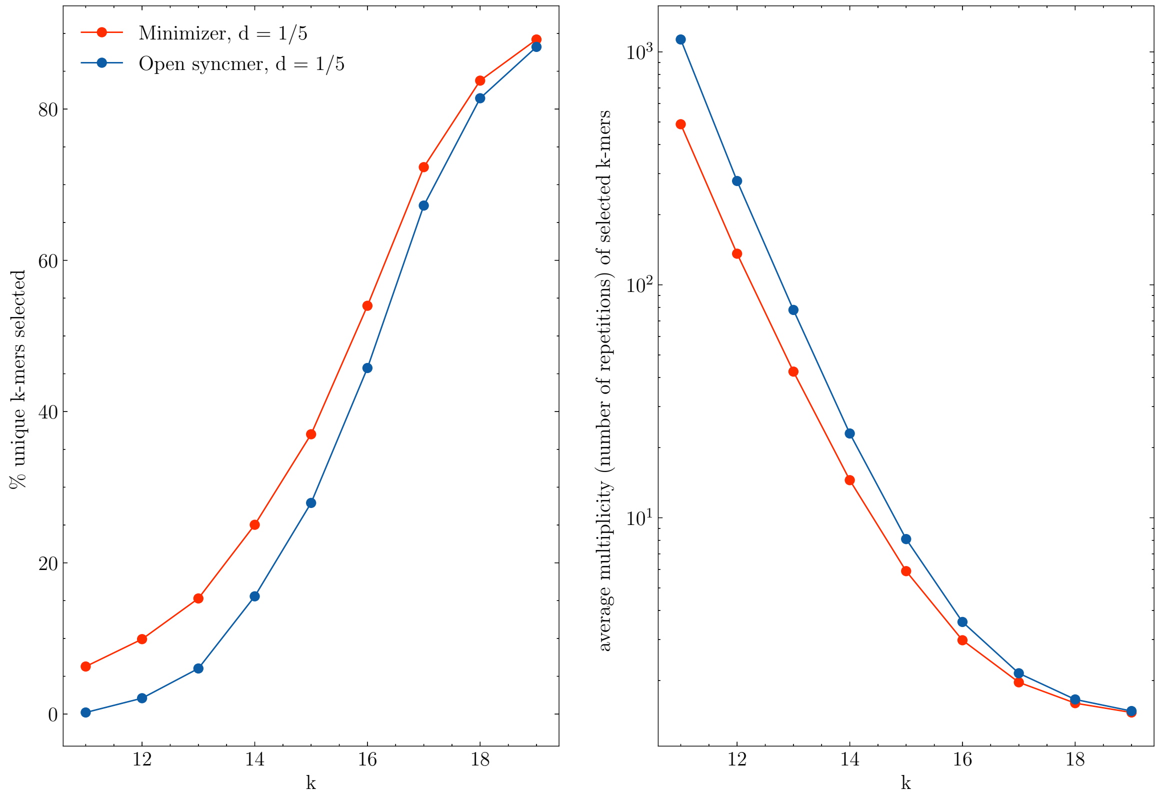


Figure 7: Left: Number of unique k-mers in the indexed set of k-mers by open syncmers versus minimizers over GRCh38. Right: Average multiplicity (number of times a k-mer repeats over the indexed set of k-mers).

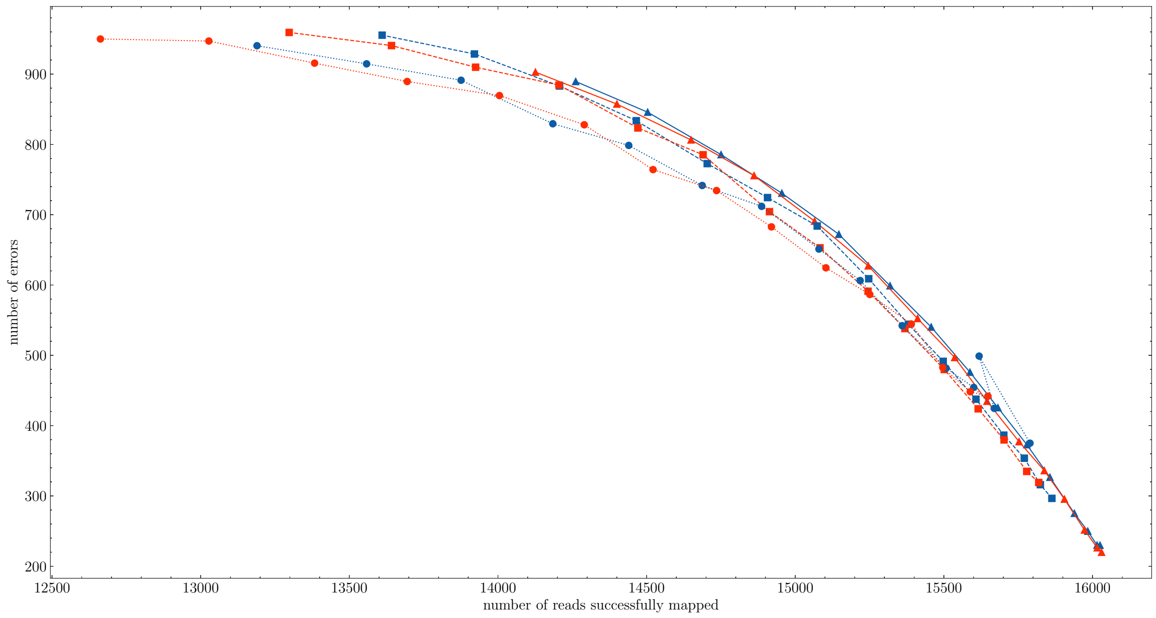


Figure 8: A larger version of the top-right subplot in Figure 5 of the main manuscript.

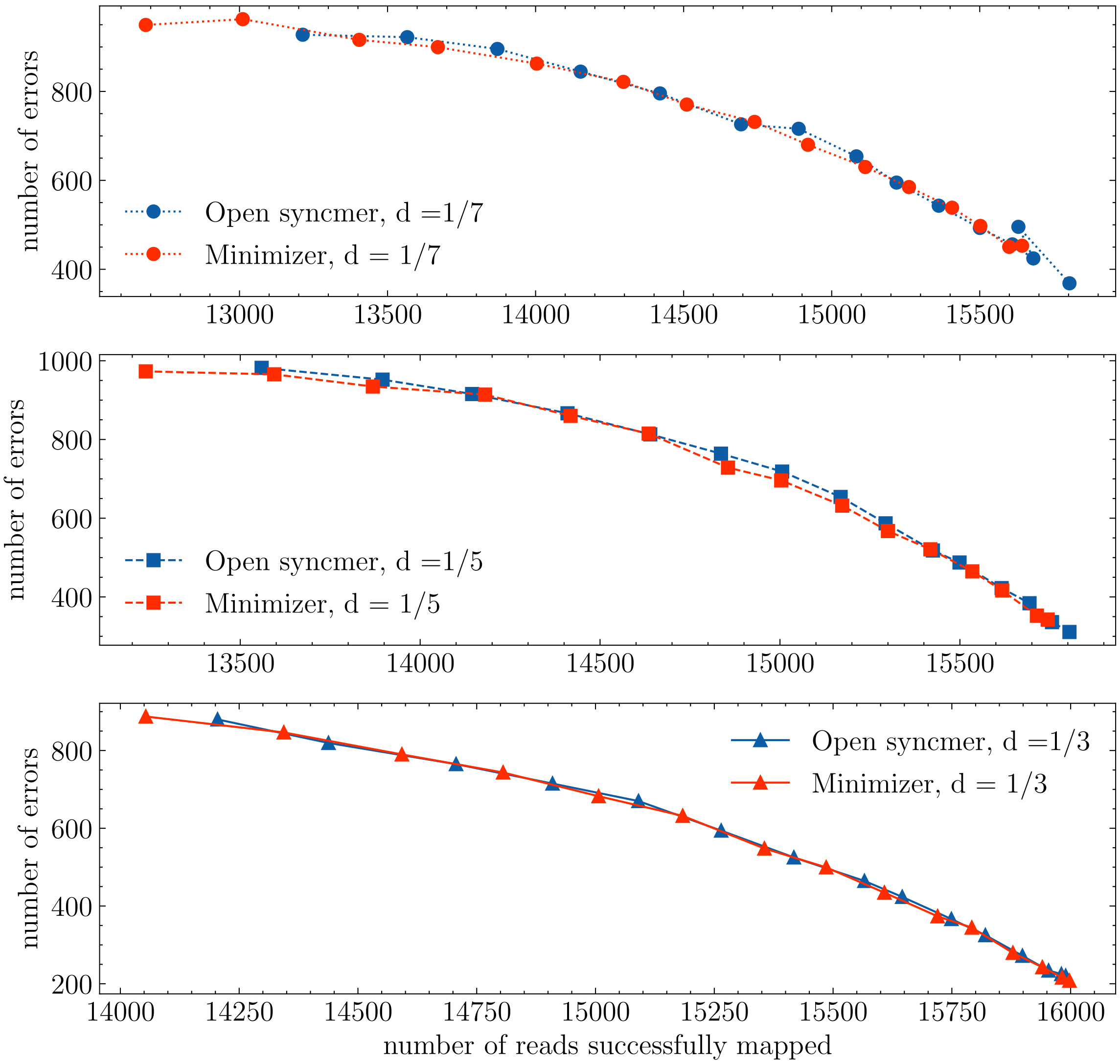


Figure 9: A larger version of the top-right subplot in Figure 5 of the main manuscript with each density plotted separately.

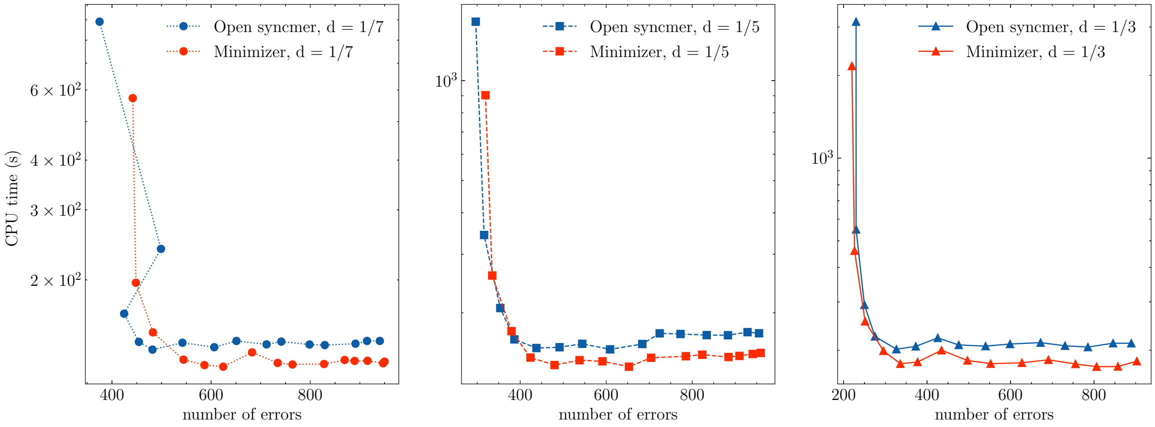


Figure 10: A larger version of the Figure 6 in the main manuscript with each density plotted separately.

# References

[1] H. Zheng, C. Kingsford, and G. Marçais, “Improved design and analysis of practical minimizers,” *Bioinformatics*, vol. 36, pp. i119–i127, July 2020.

[2] M. C. Frith, L. Noé, and G. Kucherov, “Minimally-overlapping words for sequence similarity search,” *Bioinformatics (Oxford, England)*, Dec. 2020.

[3] Y. Ono, K. Asai, and M. Hamada, “PBSIM: PacBio reads simulator—toward accurate genome assembly,” *Bioinformatics*, vol. 29, pp. 119–121, Jan. 2013.

[4] H. Li, “Minimap2: pairwise alignment for nucleotide sequences,” *Bioinformatics*, vol. 34, pp. 3094–3100, Sept. 2018.

[5] C. Jain, A. Rhie, H. Zhang, C. Chu, B. P. Walenz, S. Koren, and A. M. Phillippy, “Weighted minimizer sampling improves long read mapping,” *Bioinformatics*, vol. 36, pp. i111–i118, July 2020.
